# Parsimony and parameter estimation for mixtures of multivariate leptokurtic-normal distributions

**DOI:** 10.1007/s11634-023-00558-2

**Published:** 2023-09-27

**Authors:** Ryan P. Browne, Luca Bagnato, Antonio Punzo

**Affiliations:** 1 Department of Statistics and Actuarial Science, University of Waterloo, Waterloo, ON Canada; 2 Department of Economic and Social Sciences, Catholic University of the Sacred Heart, Milano, Italy; 3 Department of Economics and Business, University of Catania, Catania, Italy

**Keywords:** Leptokurtic-normal distribution, Majorization–minimization algorithm, Mixture models, Parsimony, 62Hxx Multivariate analysis, 62H30 Classification and discrimination; cluster analysis (statistical aspects); mixture models, 62H12 Estimation in multivariate analysis, 62G07 Density estimation

## Abstract

Mixtures of multivariate leptokurtic-normal distributions have been recently introduced in the clustering literature based on mixtures of elliptical heavy-tailed distributions. They have the advantage of having parameters directly related to the moments of practical interest. We derive two estimation procedures for these mixtures. The first one is based on the majorization-minimization algorithm, while the second is based on a fixed point approximation. Moreover, we introduce parsimonious forms of the considered mixtures and we use the illustrated estimation procedures to fit them. We use simulated and real data sets to investigate various aspects of the proposed models and algorithms.

**Supplementary Information:**

The online version contains supplementary material available at 10.1007/s11634-023-00558-2.

## Introduction

Finite mixtures of distributions are a powerful clustering tool (Titterington et al. 1985; Bouveyron et al. 2019; McNicholas 2016a). In this family, for a *d*-variate continuous random vector $\varvec{X}$, mixtures of multivariate normal distributions have received considerable attention because of their computational and theoretical convenience by assuming, in most cases, that each mixture component represents a cluster (or group) within the original data (see, e.g., Fraley and Raftery 2002 and McNicholas 2016b). The popularity of these mixtures has been further increased since the works of Banfield and Raftery (1993) and Celeux and Govaert (1995), that introduce parsimony via the eigendecomposition of the component covariance matrices. This produces a set of 14 parsimonious models that can handle various clustering scenarios and are easy to interpret.

However, in many real data applications, the tails of the component normal distributions are shorter than required. This can have a negative effect on the underlying clustering structure and also on the estimates of the component parameters (Peel and McLachlan 2000). For this reason, several mixtures of elliptical heavy-tailed distributions have been proposed in the literature. Examples are mixtures of the following multivariate distributions: *t* (Peel and McLachlan 2000; Andrews and McNicholas 2012), contaminated normal (Punzo and McNicholas 2016; Punzo et al. 2018, 2020), leptokurtic-normal (Bagnato et al. 2017, 2022), power exponential (Zhang and Liang 2010 and Dang et al. 2015), tail-inflated normal (Punzo and Bagnato 2021; Tomarchio et al. 2022) and shifted exponential normal (Punzo and Bagnato 2020 and Tomarchio et al. 2022). Among them, mixtures of multivariate leptokurtic-normal (MLN) distributions (refer to Sect. 2) have the advantage to be characterized by parameters directly related to the moments of practical interest, especially in each cluster.

For maximum likelihood (ML) estimation of the parameters of MLN mixtures, Bagnato et al. (2017) used the EM algorithm; in particular, they adopted the general-purpose optimizer optim(), provided in R, to update the component-wise parameters in each M-step of the algorithm. Browne (2022) introduced a fixed point (FP) algorithm and showed improvements in terms of computational time. However, the FP algorithm is not guaranteed to be monotonic although Browne (2022) provided some evidence of this through simulations.

In Sect. 4, we explore parsimonious forms of MLN mixtures by setting some parameters to be equal across components. For ML estimation we derive two algorithms, one based on the MM principle (Lange et al. 2000; Hunter and Lange 2004), in Sect. 5, and the second one based on the FP algorithm from Browne (2022), in Sect. 6. The MM algorithm is based on a quadratic function obtained by using a bound on the second derivative of the log-density. We derive this bound and show connection between the MM and FP procedures in Sect. 3.

The remaining part of the paper is structured as follows. In Sect. 7, we first use simulated data to compare the two proposed algorithms in terms of computational burden and solutions obtained (Sect. 7.1). Then, in Sect. 7.2, we use real data with the same aim as for Sect. 7.1, but also to show the existence of real data situations where a parsimonious variant of the general model can provide a better description of the data. In Sect. 8, we describe the results of a further simulation study aiming to investigate aspects such as: the evaluation of the asymptotic properties of the ML estimator (Section E.1 in the supplementary material), model selection via the BIC (Sect. 8.1), and the behavior of the parsimonious MLN mixtures under different data generating models (Sect. 8.2). We give a final overview of the results obtained in this paper in Sect. 9.

A supplementary material is also available online where we provide: an overview about the notation and the main functions used herein (Section A), useful details for the implementation of the proposed approaches (Sections B and C), and some tables, figures, and details related to the simulation studies discussed in Sects. 7.1 (Section D) and 8 (Section E). Section F of the supplementary material provides an additional simulation study to investigate the viability of the estimation procedures on large data sets and sampling variability of the estimation procedure.

## Finite mixtures of MLN distributions

The MLN distribution—with mean vector $\varvec{\mu }$, covariance matrix $\varvec{\Sigma }$, and excess kurtosis $\beta$—was introduced by Bagnato et al. (2017) and has the following joint probability density function (pdf)

1 $$\begin{aligned} f\left( \varvec{x}\mid \varvec{\mu }, \varvec{\Sigma }, \beta \right) = \left\{ 1 +\beta g\left[ \left( \varvec{x}-\varvec{\mu }\right) ^\top \varvec{\Sigma }^{-1}\left( \varvec{x}-\varvec{\mu }\right) \right] \right\} \phi \left( \varvec{x}\mid \varvec{\mu }, \varvec{\Sigma }\right) , \end{aligned}$$

where $\phi \left( \varvec{x}\mid \varvec{\mu }, \varvec{\Sigma }\right)$ denotes joint pdf of the multivariate normal (MN) distribution with mean $\varvec{\mu }$ and covariance matrix $\varvec{\Sigma }$,

2 $$\begin{aligned} g\left( r\right) = \frac{r^2 - 2(d+2) r + d(d+2)}{8d(d+2)}, \end{aligned}$$

and

$$\begin{aligned} \beta \in \left[ 0, \frac{4d(d + 2)}{d + 4} \right] . \end{aligned}$$

The MLN distribution is obtained by applying a multivariate Gram-Charlier expansion to the MN distribution and has one additional parameter, $\beta$, that, as already said above, controls the excess kurtosis.

For model-based clustering purposes, Bagnato et al. (2017) also proposed and illustrated finite mixtures of MLN distributions with joint pdf

3 $$\begin{aligned} p\left( \varvec{x}\mid \varvec{\theta }\right) = \sum _{j=1}^k \pi _j f\left( \varvec{x}\mid \varvec{\mu }_j, \varvec{\Sigma }_j, \beta _j\right) , \end{aligned}$$

where $\pi _j>0$, $\sum _{j=1}^k \pi _j =1$, and $\varvec{\theta }$ contains all parameters.

Bagnato et al. (2017) proposed (1) to model data with excess kurtosis and (3) as a robust model based clustering approach. Browne (2022) showed that finite mixtures of MLN distributions are identifiability when $(\varvec{\mu }_j,\varvec{\Sigma }_j)$ are different; he also suggested that, if $\left( \varvec{\mu }_j,\varvec{\Sigma }_j\right) = \left( \varvec{\mu }_t,\varvec{\Sigma }_t\right)$ for some $j\ne t$, then these two components are to be combined with $\beta ^{\star } = \pi _j \beta _j + \pi _t \beta _t$.

## ML estimation: surrogate functions for MM and FP procedures

Herein, we derive the key functions for the MM and FP procedures used to ML estimates of the parameters of the MLN distribution. First, we find a bound for the Hessian. To derive a bound we begin by rewriting the pdf in (1) as a function of both the standardized vector $\varvec{z}= \varvec{\Sigma }^{-1/2}\left( \varvec{x}-\varvec{\mu }\right)$ and the squared Mahalanobis distance $r = \varvec{z}^\top \varvec{z}=\left( \varvec{x}-\varvec{\mu }\right) ^\top \varvec{\Sigma }^{-1}\left( \varvec{x}-\varvec{\mu }\right)$ as

4 $$\begin{aligned} u\left( r\right) := \log f\left( r \right) = \log f\left( \varvec{z}^\top \varvec{z}\right) = - \frac{d}{2} \log \left( 2\pi \right) - \log \left| \varvec{\Sigma }\right| + \log \left[ 1 + \beta g\left( r\right) \right] - \frac{r}{2}. \end{aligned}$$

Browne (2022) used this form to show that the log-density is unimodal by finding a bound on the first derivative,

$$\begin{aligned} u'\left( r\right) := \frac{d }{d r} \log f\left( r\right) = \frac{ \beta g'\left( r\right) }{1 + \beta g\left( r\right) } - \frac{1}{2}. \end{aligned}$$

Here, we expand on this by finding a bound on the Hessian. Using the chain rule we can obtain the Hessian and we find

$$\begin{aligned} \frac{d^2 \log f( \varvec{z}) }{d \varvec{z}d \varvec{z}^\top } = \frac{d^2 \log f( r ) }{d r } \; 4 \varvec{z}\varvec{z}^\top + 2 \frac{d \log f( r ) }{d r } \varvec{I}_d \preceq \left( \frac{d^2 \log f( r ) }{d r } 4 r + 2 \frac{d \log f( r ) }{d r } \right) \varvec{I}_d, \end{aligned}$$

where $\varvec{I}_d$ denotes the $d\times d$ identity matrix and $\varvec{A}\preceq \varvec{B}$ means that $\varvec{B}- \varvec{A}$ is positive definite. This bound depends on the following univariate function

5 $$\begin{aligned} h\left( r\right) := \frac{d^2 \log f\left( r\right) }{d r } \; 4 r + 2 \frac{d \log f\left( r\right) }{d r } = \frac{ \beta g''\left( r\right) }{ 1 + \beta g\left( r\right) } \; 4r - \left[ \frac{ \beta g'\left( r\right) }{ 1 + \beta g\left( r\right) } \right] ^2 \; 4r + 2 \frac{ \beta g'\left( r\right) }{ 1 + \beta g\left( r\right) }. \end{aligned}$$

We can then find the maximum and minimum of $h(\cdot )$ by taking the derivative with respect to *r*. Setting the derivative equal to zero we find that the stationary points are roots to the following depressed quartic polynomial

$$\begin{aligned} -3d(d + 2)^2 (\beta + 8)(\beta d + 4\beta - 8d) + 8\beta (d + 2)^2 (\beta d - \beta + 12 d) \; r + 6 \beta (d + 2) (\beta -4d ) \; r^2 + \beta ^2 \; r^4 =0. \end{aligned}$$

**Fig. 1:**
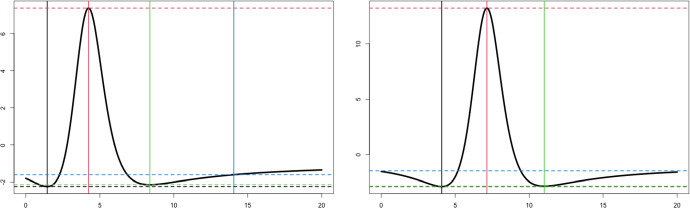
Examples of $h\left( r\right)$ function along with the stationary points and bounds with $\beta = 4d(d+2)/(d+4)$. Left panel has $d=2$ and right panel has $d=5$

Then, maximum and minimum are obtained by evaluating $h(\cdot )$ at the four roots. These roots and bounds are illustrated in Fig. 1 for two different values of *d* (2 and 5).

Of particular interest is the lower bound *M*, as it can be used to construct a quadratic minorizer function. We can obtain it simply evaluating $h(\cdot )$ at the four roots, say $r_1$, $r_2$, $r_3$, and $r_4$, i.e.

6 $$\begin{aligned} M = \bigg | \min \left\{ h(r_1), h(r_2), h(r_3), h(r_4) \right\} \bigg |. \end{aligned}$$

Note, we apply the absolute value so that the quadratic minorizer has a particular form. Also, the value *M* only depends on $\beta$ and *d*.

Applying this bound, we can construct the following quadratic minorizer, using the expansion point $\varvec{z}_0$,

7 $$\begin{aligned} \begin{aligned} \log f\left( \varvec{z}^\top \varvec{z}\right)&\ge \log f\left( \varvec{z}_0^\top \varvec{z}_0 \right) + \nabla \log f\left( \varvec{z}^\top \varvec{z}\right) \left( \varvec{z}- \varvec{z}_0 \right) - \frac{M}{2} \left( \varvec{z}- \varvec{z}_0 \right) ^\top \left( \varvec{z}- \varvec{z}_0 \right) \\&\ge C_0 + \left[ 2 u'\left( \varvec{z}_0^\top \varvec{z}_0 \right) + M \right] \varvec{z}_0^\top \varvec{z}- \frac{M}{2} \varvec{z}^\top \varvec{z}, \\ \end{aligned} \end{aligned}$$

where $C_0$ depends on $\varvec{z}_0$. This quadratic minorizer is used as a starting point for the MM algorithm presented in Sect. 6.

Alternatively, the following quadratic surrogate function can be constructed

8 $$\begin{aligned} \begin{aligned} \log f\left( \varvec{z}^\top \varvec{z}\right)&\approx \log f\left( \varvec{z}_0^\top \varvec{z}_0 \right) + \left. \frac{d}{d r} \log f\left( r \right) \right| _{r= \varvec{z}_0^\top \varvec{z}_0 } \; \left( \varvec{z}^\top \varvec{z}- \varvec{z}_0^\top \varvec{z}_0 \right) \\&\approx \log f\left( \varvec{z}_0^\top \varvec{z}_0 \right) + u'\left( \varvec{z}_0^\top \varvec{z}_0 \right) \left( \varvec{z}^\top \varvec{z}- \varvec{z}_0^\top \varvec{z}_0 \right) . \end{aligned} \end{aligned}$$

This approximation was considered by Browne (2022) and is used as a starting point for the FP algorithm presented in Sect. 6. Browne (2022) showed that even though this surrogate function is not minorizer, an algorithm can be constructed to yield monotonic log-likelihood values. Monotonicity of the log-likelihood values was shown through simulations.

**Fig. 2:**
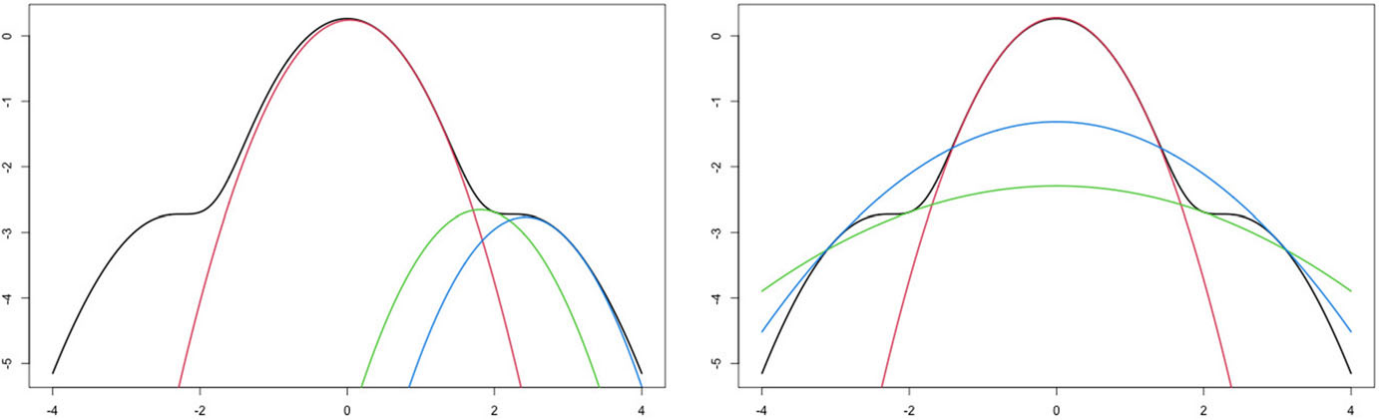
Left panel: The log density, $\log f(z^2)$, along with three quadratic minorizers based on (7) using the expansion points $z_0=1,2,3$. Right panel: The log density, $\log f(z^2)$, along with three quadratic surrogate functions based on (8) using the expansion points $z_0=1,2,3$. Both figures are based on $d=1$ and $\beta = 4d(d+2)/(d+4)$

Figure 2 illustrates the functions given in (7) and (8), when $d=1$ and $\beta = 4d(d+2)/(d+4)$ when using expansion points equal to $z_0=1,2,3$.

## Parsimonious finite mixtures

Here, we introduce parsimonious forms of the finite mixture model in (3). Similar to Fraley and Raftery (2002) and Celeux and Govaert (1995), we consider the following 3-term eigendecomposition of the covariance matrix

9 $$\begin{aligned} \varvec{\Sigma }_j = \lambda _j \varvec{\Gamma }_j \varvec{\Psi }_j \varvec{\Gamma }_j^\top , \end{aligned}$$

where $\lambda _j=|\varvec{\Psi }_j|^{1/d}$, $\varvec{\Gamma }_j$ is a $d \times d$ orthogonal matrix of the eigenvectors of $\varvec{\Sigma }_j$, $\varvec{\Psi }_j$ is the $d \times d$ diagonal matrix with the scaled eigenvalues of $\varvec{\Sigma }_j$ (such that $\left| \varvec{\Psi }_j \right| = 1$) located on the main diagonal. The decomposition in (9) has some useful practical interpretations. From a geometric point of view, $\lambda _j$ determines the volume, $\varvec{\Gamma }_j$ governs the orientation, and $\varvec{\Psi }_j$ denotes the shape of the *j*th cluster. From a statistical point of view, as well-documented in Fraley and Raftery (2002), Celeux and Govaert (1995), Greselin and Punzo (2013), Bagnato and Punzo (2021), and Punzo and Bagnato (2022), the columns of $\varvec{\Gamma }_j$ govern the orientation of the principal components (PCs) of the *j*th cluster, the diagonal elements in $\varvec{\Psi }_j$ are the normalized variances of these PCs, and $\lambda _j$ can be meant as the overall volume of the scatter in the space spanned by the PCs of the *j*th cluster.

By setting the three parameters be equal, vary or fixed across groups (See Celeux and Govaert 1995), the decomposition in (9) yields fourteen different models. These model are denoted by three letters (Fraley et al. 2015; Scrucca et al. 2016; Pocuca et al. 2022). The MLN model has an additional parameter $\beta _j$ which can be equal or vary across groups. This results in twenty eight possible models. This family of models is represented using a 4-letter identifier of the type $X_1X_1X_3X_4$, where:

1. $X_1=E$ if $\lambda _j=\lambda$ or $X_1=V$ if $\lambda _j$ is varying across groups;
2. $X_2=I$ if $\varvec{\Psi }_j=\varvec{I}_d$, $X_2=E$ if $\varvec{\Psi }_j=\varvec{\Psi }$ or $X_2=V$ if $\varvec{\Psi }_j$ is varying across groups;
3. $X_3=I$ if $\varvec{\Gamma }_j=\varvec{I}_d$, $X_3=E$ if $\varvec{\Gamma }_j=\varvec{\Gamma }$ or $X_3=V$ if $\varvec{\Gamma }_j$ is varying across groups;
4. $X_4=E$ if $\beta _j = \beta$ or $X_4=V$ if $\beta _j$ is varying across groups.

Just as an example, VEIE indicates $\varvec{\Sigma }_j = \lambda _j \varvec{\Psi }$ and $\beta _j=\beta$. However, we will also use a 3-letter identifier $X_1X_1X_3$ when $\beta _j$, $j=1,\ldots ,d$, will be considered as fixed.

### ML estimation

Similar to Bagnato et al. (2017) and Browne (2022), we apply the expectation-maximization (EM) algorithm (Dempster et al. 1977) to obtain ML estimates. In the context of finite mixture models, we introduce the component memberships as missing data for each observation $\varvec{x}_i$ in the sample. We represent the missing data using an indicator variable, denoted by $w_{ij}$, such that $w_{ij}=1$ if observation *i* was generated by component *j* and $w_{ij}=0$ otherwise. At iteration *q*, the expected value of the missing data given the observed data $\varvec{x}_i$ and the current parameter values (at iteration *q*) is

$$\begin{aligned} w_{ij}^{(q)} = \frac{ \pi _j^{(q)} f\left( \varvec{x}_i \mid \varvec{\mu }_j^{(q)}, \varvec{\Sigma }_j^{(q)}, \beta _j^{(q)} \right) }{ \displaystyle \sum\nolimits_{h=1}^k \pi _h^{(q)} f\left( \varvec{x}_i \mid \varvec{\mu }_h^{(q)}, \varvec{\Sigma }_h^{(q)}, \beta _h^{(q)} \right) }. \end{aligned}$$

Using these expected values, we can form the expected complete log-likelihood

10 $$\begin{aligned} C + \sum _{i=1}^n \sum _{j=1}^k w_{ij}^{(q)} \left\{ \log \pi _j - \frac{ r_{ij} }{2} + \log \left[ 1 + \beta _j g\left( r_{ij} \right) \right] \right\} , \end{aligned}$$

where $r_{ij} = \left( \varvec{x}_i -\varvec{\mu }_j\right) ^\top \varvec{\Sigma }_j^{-1}\left( \varvec{x}_i -\varvec{\mu }_j\right)$ and *C* does not depend on the parameters.

The M-step involves maximizing the expected complete log-likelihood in (10). The update for $\pi _j$ is $\pi _j^{(q+1)} = n_j^{(q)}/n$, where $n_j^{(q)} = \sum _{i=1}^n w_{ij}^{(q)}$. The update for $\beta _j$, when equal and varying across components, is given in Sect. 4.2. Then, for $(\varvec{\mu }_1, \varvec{\Sigma }_1, \ldots , \varvec{\mu }_k, \varvec{\Sigma }_k)$ we consider two alternative methods for estimation; a MM algorithm and a FP algorithm.

The MM algorithm is based the minorizer derived in (7), while the FP algorithm is based on the surrogate function in (8).

Section 5 gives an overview of the MM algorithm but all the technical derivations for each model are quite long, so we provide the details in Section B of the supplementary material. The FP algorithm updates are shown in Sect. 6. The FP algorithm have form related to Celeux and Govaert (1995) and as such we can use formulae similar to Celeux and Govaert (1995) and Browne and McNicholas (2014a) to construct updates for all the eigendecomposed models.

### Updating $\beta _j$ or $\beta$

#### Varying $\beta _j$

For the parameter $\beta _j$, Bagnato et al. (2017) maximizes the weighted log-likelihood with respect to a transformed variable $\eta _j= \log [ \beta _j /(\beta _{\text {max}} - \beta _j )]$, where $\beta _{\text {max}}$ is the largest value $\beta _j$ can be. However, Browne (2022) showed that the log-likelihood (10), as a function of $\beta _j$, is self concordant. Self concordant functions yield a bound on the number of Newton steps required to minimize a function (for more details see Boyd and Vandenberghe 2006).

An unconstrained Newton step for $\beta _j$ is

$$\begin{aligned} \beta _j^{(q+1)} = \beta _j^{(q)} + \frac{ \displaystyle \sum\nolimits_{i=1}^n w_{ij}^{(q)} \gamma _i\left( \varvec{\mu }_j^{(q)}, \varvec{\Sigma }_j^{(q)}, \beta _j^{(q)} \right) }{ \displaystyle \sum\nolimits_{i=1}^n w_{ij}^{(q)} \left[ \gamma _i\left( \varvec{\mu }_j^{(q)}, \varvec{\Sigma }_j^{(q)}, \beta _j^{(q)} \right) \right] ^2 }, \end{aligned}$$

where

$$\begin{aligned} \gamma _i\left( \varvec{\mu }, \varvec{\Sigma }, \beta \right) = \frac{ \beta g\left[ \left( \varvec{x}_i -\varvec{\mu }\right) ^\top \varvec{\Sigma }^{-1}\left( \varvec{x}_i -\varvec{\mu }\right) \right] }{1 + \beta g\left[ \left( \varvec{x}_i -\varvec{\mu }\right) ^\top \varvec{\Sigma }^{-1}\left( \varvec{x}_i -\varvec{\mu }\right) \right] }. \end{aligned}$$

We apply this update while maintaining that $\beta _j$ stays within the range of unimodality. Alternatively, one could apply an univariate numerical optimization or incorporate backtracking with Newton’s method.

#### Equal $\beta _j = \beta$

Let $\beta _j$ be equal across components, i.e., $\beta _j=\beta$. Since self concordance is preserved under summation and affine transformation, the log-likelihood is again concordant. Update for $\beta$, based on an unconstrained Newton step, is given by

$$\begin{aligned} \beta ^{(q+1)} = \beta ^{(q)} + \frac{\displaystyle \sum\nolimits_{i=1}^n \sum\nolimits_{j=1}^k w_{ij}^{(q)} \gamma _i\left( \varvec{\mu }_j^{(q)}, \varvec{\Sigma }_j^{(q)}, \beta ^{(q)} \right) }{\displaystyle \sum\nolimits_{i=1}^n \sum\nolimits_{j=1}^k w_{ij}^{(q)} \left[ \gamma _i\left( \varvec{\mu }_j^{(q)}, \varvec{\Sigma }_j^{(q)}, \beta ^{(q)} \right) \right] ^2 }. \end{aligned}$$

## MM algorithm for updating $\varvec{\mu }_j$ and $\varvec{\Sigma }_j$

Applying $\beta _j$ to (6) we obtain $M_j$ and then using (7), we can obtain the following minorizer function

$$\begin{aligned} \begin{aligned}&C - \frac{n_j^{(q)}}{2}\log \left| \varvec{\Sigma }_j \right| + {{\,\textrm{Tr}\,}}\left\{ \varvec{\Sigma }^{-1/2}_j \sum\nolimits_{i=1}^n w_{ij}^{(q)} \left[ 2 u'\left( r_{ij}^{(q)} \right) + M_j \right] \left( \varvec{x}_i - \varvec{\mu }_j \right) \left( \varvec{z}_{ij}^{(q)}\right) ^\top \right\} \\&\qquad \qquad \qquad \qquad \qquad - \frac{M_j}{2} {{\,\textrm{Tr}\,}}\left[ \varvec{\Sigma }^{-1}_j \sum\nolimits_{i=1}^n w_{ij}^{(q)} \left( \varvec{x}_i - \varvec{\mu }_j \right) \left( \varvec{x}_i - \varvec{\mu }_j \right) ^\top \right] , \end{aligned} \end{aligned}$$

where *C* does not depend on the parameters, $r_{ij}^{(q)} = \left( \varvec{z}_{ij}^{(q)} \right) ^\top \varvec{z}_{ij}^{(q)}$ and $\varvec{z}_{ij}^{(q)} = \left( \varvec{\Sigma }_j^{(q)}\right) ^{-1/2} \left( \varvec{x}_i - \varvec{\mu }_j^{(q)} \right)$. Taking the derivative, and solving, yields the following update for $\varvec{\mu }_j$

$$\begin{aligned} \varvec{\mu }_j^{(q+1)} = \overline{\varvec{x}}_j^{(q)} - \frac{1}{M_j n_j^{(q)}} \sum _{i=1}^n w_{ij}^{(q)} \left[ 2 u'\left( r_{ij}^{(q)} \right) + M_j \right] \left[ \varvec{\Sigma }_j^{(q)} \right] ^{1/2} \left( \varvec{z}_{ij}^{(q)}\right) ^\top , \end{aligned}$$

where

$$\begin{aligned} \overline{\varvec{x}}_j^{(q)} = \frac{1}{n_j^{(q)}}\sum _{i=1}^n w_{ij}^{(q)}\varvec{x}_i. \end{aligned}$$

The minorizer, in terms of the covariance parameters, with updated $\varvec{\mu }_j^{(q+1)}$, is

11 $$\begin{aligned} \sum _{j=1}^k\left[ - \frac{n_j^{(q)}}{2}\log \left| \varvec{\Sigma }_j \right| + {{\,\textrm{Tr}\,}}\left( \varvec{\Sigma }^{-1/2}_j \varvec{A}_j \right) - \frac{1}{2} {{\,\textrm{Tr}\,}}\left( \varvec{\Sigma }^{-1}_j \varvec{B}_j \right) \right] , \end{aligned}$$

where

$$\begin{aligned} \varvec{A}_j = \sum _{i=1}^n w_{ij}^{(q)} \left[ 2 u'\left( r_{ij}^{(q)} \right) + M_j \right] \left( \varvec{x}_i - \varvec{\mu }_j^{(q+1)} \right) \left( \varvec{z}_{ij}^{(q)}\right) ^\top \end{aligned}$$

and

$$\begin{aligned} \varvec{B}_j = M_j \sum _{i=1}^n w_{ij}^{(q)} \left( \varvec{x}_i - \varvec{\mu }_j^{(q+1)} \right) \left( \varvec{x}_i - \varvec{\mu }_j^{(q+1)} \right) ^\top . \end{aligned}$$

Equation (11) is the starting point for all the eigendecomposed models.

The technical derivations for each model are quite long, so we provide the details for each model in Section B of the supplementary material. When considering a model, we repeatedly use reparameterization and variety of methods to obtain updates for the parameters. For example, for the VVV model we let $\varvec{\Xi }_j = \varvec{\Sigma }_j^{-1/2}$ then use the algebraic Riccati equation (for review, see Wonham 1968) to obtain an unique symmetric positive definite update based method from Laub (1979). Estimation of $\varvec{\Gamma }$ with the constraint $\varvec{\Gamma }^\top \varvec{\Gamma }= \varvec{I}_d$ has been considered by Browne and McNicholas (2014b) and Absil et al. (2008), but here we will follow Browne and McNicholas (2014a) and Kiers (2002).

## FP algorithm for updating $\varvec{\mu }_j$ and $\varvec{\Sigma }_j$

When we are in the generic $j$th component, using (8), an alternative surrogate function for the expected complete log-likelihood (10) is

$$\begin{aligned} C - \frac{n_j^{(q)}}{2}\log \left| \varvec{\Sigma }_j \right| - \frac{M}{2} {{\,\textrm{Tr}\,}}\left[ \varvec{\Sigma }^{-1}_j \sum _{i=1}^n w_{ij}^{(q)} \kappa _i\left( \varvec{\mu }_j^{(q)}, \varvec{\Sigma }_j^{(q)}, \beta _j^{(q)} \right) \left( \varvec{x}_i - \varvec{\mu }_j \right) \left( \varvec{x}_i - \varvec{\mu }_j \right) ^\top \right] , \end{aligned}$$

where

$$\begin{aligned} \kappa _i\left( \varvec{\mu }, \varvec{\Sigma }, \beta \right) = 1 - 2 \frac{ \beta g'\left[ \left( \varvec{x}_i -\varvec{\mu }\right) ^\top \varvec{\Sigma }^{-1}\left( \varvec{x}_i -\varvec{\mu }\right) \right] }{1 + \beta g\left[ \left( \varvec{x}_i -\varvec{\mu }\right) ^\top \varvec{\Sigma }^{-1}\left( \varvec{x}_i -\varvec{\mu }\right) \right] }. \end{aligned}$$

Note that $\kappa _i(\cdot )$ is bounded to the interval [0, 1]. Taking the derivative, and solving, yields the following update for $\varvec{\mu }_j$

12 $$\begin{aligned} \varvec{\mu }_j^{(q+1)} = \frac{ \displaystyle \sum\nolimits_{i=1}^n w_{ij}^{(q)} \kappa _i\left( \varvec{\mu }_j^{(q)}, \varvec{\Sigma }_j^{(q)}, \beta _j^{(q)} \right) \varvec{x}_i }{ \displaystyle \sum\nolimits_{i=1}^n w_{ij}^{(q)} \kappa _i\left( \varvec{\mu }_j^{(q)}, \varvec{\Sigma }_j^{(q)}, \beta _j^{(q)} \right) }. \end{aligned}$$

Then, using the updated $\varvec{\mu }_j$, the update for $\varvec{\Sigma }_j$ is based on the following quantity

$$\begin{aligned} \varvec{R}_j^{(q)} = \frac{1}{n_{j}^{(q)}} \sum _{i=1}^n \left[ w_{ij}^{(q)} \kappa _i\left( \varvec{\mu }_j^{(q+1)}, \varvec{\Sigma }_j^{(q)}, \beta _j^{(q)} \right) \right] \left( \varvec{x}_i -\varvec{\mu }_j^{(q+1)} \right) \left( \varvec{x}_i -\varvec{\mu }_j^{(q+1)} \right) ^\top . \end{aligned}$$

Browne (2022) noticed using $\varvec{R}_j^{(q)}$ to update $\varvec{\Sigma }_j$ can cause fluctuations in the log-likelihood values; so, instead, the author suggests using a weighted average

13 $$\begin{aligned} \varvec{S}^{(q)}_j = \left( 1-a^{(q)}\right) \varvec{R}_j^{(q)} + a^{(q)} \varvec{R}_j^{(q)} \quad \text{ where } \quad a^{(q)} = \frac{ 1}{ 1 + \beta ^{(q)} \frac{ d+4}{4d (d + 2)} }, \end{aligned}$$

so that the weight is 1 when $\beta ^{(q)} = 0$ and the weight is 1/2 when $\beta ^{(q)}$ is equal to the largest possible value of $4d (d + 2)/(d+4)$. In Section C of the supplementary material we explore different values for the weight.

We use $\varvec{S}^{(q)}_1, \ldots , \varvec{S}^{(q)}_k$ and follow Celeux and Govaert (1995) to update all the eigendecomposed models, except for VVE and EVE where we follow Browne and McNicholas (2014a). Just as an example, for the VVV (or varying $\varvec{\Sigma }_j$) model, the update is $\varvec{\Sigma }_j^{(q+1)} = \varvec{S}^{(q+1)}_j$, and for the EEE ($\varvec{\Sigma }_j = \varvec{\Sigma }$) model the update is

$$\begin{aligned} \varvec{\Sigma }^{(q+1)} =\sum _{j=1}^k \pi _j^{(q+1)} \varvec{S}^{(q)}_j. \end{aligned}$$

Using the updates (12) and (13) does not guarantee an increase in the log-likelihood. However, Browne (2022) noted that the sequence of log-likelihoods generated from the EM exhibited monotonicity and no fluctuations in the log-likelihood values. Section C of the supplementary material illustrates fluctuations in the sequence of likelihood values when $a=1$ and $\beta =4d(d+2)/(d+4$ and investigates a range of weights. Similar to Browne (2022), when using (12) and (13), the log-likelihood sequences obtained from the simulation studies in Sects. 7 and 8 exhibited monotonicity. This provides additional evidence towards a monotonicity property for this FP procedure.

Since the updating procedure for the weighted log-likelihood can be viewed as a generalized EM algorithm, convergence criteria, such as the Aitken acceleration (see Böhning et al. 1994 and Lindsay 1995), can be applied.

## Analyses on simulated and real data

Using the R package Browne et al. (2023), we simulated and real data to compare the MM and FP algorithms in terms of computational time and solutions obtained when using: (1) the random starting values and (2) the Aitken acceleration (Aitken 1926, Böhning et al. 1994 and Lindsay 1995) as stopping criterion, using a tolerance equal to $10^{-6}$. The computational time is calculated using the wall-clock time on an iMac with 4 GHz Quad-Core Intel Core i7. Real data are also used to show the importance of considering parsimonious variants of the general, unconstrained, MLN mixture model (VVVV; Sect. 7.2). Always in this section, we adopt the Bayesian information criterion (BIC; Schwarz 1978) for model selection and the adjusted Rand index (ARI; Hubert and Arabie 1985) for clustering/classification assessment. Note that, in the formulation used herein, the BIC values are multiplied by $-1$; this means that, in analogy with the log-likelihood, this criterion needs to be maximized.

### Simulated data: comparing MM and FP algorithms

Here, we compare the MM and FP algorithms on simulated data sets while varying the number of variables and covariance model. We hold $\beta _j$ fixed and equal to $4d(d + 2)/(d + 4)$ since both procedures use the same updates for this parameter. So, we only focus on the updates for $\varvec{\mu }_j$ and $\varvec{\Sigma }_j$, $j=1,\ldots ,k$. We assess the average computational time for a fixed number of iterations, the total time to convergence, and the number of iterations at convergence. We generate $n=500$ observations from a two component ($k=2$) MLN mixture model with parameters equal to

14 $$\begin{aligned}{} & {} (\pi _1,\pi _2) = \left( \frac{1}{2}, \frac{1}{2} \right) , \; \left\{ \varvec{\mu }_1, \varvec{\mu }_2\right\} = \left\{ - 5 \textbf{1}_d, +5 \textbf{1}_d \right\} , \; \varvec{\Sigma }_1 = \varvec{\Sigma }_2 = \varvec{I}_d, \; \text{ and } \nonumber \\{} & {} \quad \beta _1 = \beta _2 = \frac{4d(d + 2)}{(d + 4)}. \end{aligned}$$

Both algorithms use the same initial value, which is generated randomly. We varied the number of variables *d* to be 2, 8, or 16. We compare the computational time, and the log-likelihood values at convergence, from fitting a $k=2$ component MLN mixture model. We used 100 replications to reduce simulation error, i.e., differences due to a particular data set.

As an initial comparison, we evaluate the computational time for each procedure when performing 100 EM iterations. Table 1 reports the ratio of computational times (MM over FP) and indicates the FP algorithm is faster than the numerical optimization procedures except for the models with covariance equal to VVE and EVE. This is due to the FP algorithm requiring the eigenvalues from a positive definite matrix whereas the MM algorithm does not.

**Table 1:** Ratio of computational times (MM over FP), for 100 iterations, while varying the number of variables *d* and the covariance structure

*d*	EEE	VVV	VEE	EVV	EEV	VEV	EVI	EII	VEI	VVI	EEI	VII	VVE	EVE
2	1.6	1.6	1.5	1.2	1.4	1.2	1.4	1.3	1.3	1.3	1.2	1.2	0.7	0.8
8	1.5	1.5	1.4	1.3	1.4	1.3	1.5	1.4	1.4	1.4	1.4	1.4	0.8	0.8
16	2.0	1.9	1.8	1.8	1.7	1.6	1.5	1.4	1.4	1.4	1.4	1.4	1.0	0.8

The second comparison quantifies the computational time, the total number of iterations and the log-likelihood values at convergence. Table 2 displays the average computational time and iterations from the FP and MM algorithms when $d=2$ or 8. The results in the case $d=16$ are given in Section D of the supplementary material. The tables also show the mean difference in the log-likelihoods, say $\hat{l}_{\max }-\hat{l}_{\text {FP}}$ and $\hat{l}_{\max }- \hat{l}_{\text {MM}}$, where $\hat{l}_{\max }=\max \left\{ \hat{l}_{\text {FP}}, \hat{l}_{\text {MM}} \right\}$ and $\hat{l}_{\text {FP}}$ and $\hat{l}_{\text {MM}}$ are the log-likelihood values obtained at convergence for the FP and MM algorithms.

When the procedures converge, they tend to have roughly the same number of iterations and log-likelihood values. However, for some models, the MM algorithm reaches the maximum number of iterations. This indicates we might want to use a stricter convergence criterion for the MM procedure. Interestingly, EVE and VVE models had slower computational time for 100 iterations, but here we see that requiring fewer iterations yields a faster time to convergence.

**Table 2:** Average computational time and number of iterations until convergence (with the maximum number of iterations fixed to 1000), for each estimation algorithm over 100 replications

	$d=2$, Mean from (FP, MM) of						$d=8$, Mean from (FP, MM) of					
	Time		Iterations		$\hat{l}_{\max } -\hat{l}$		Time		Iterations		$\hat{l}_{\max } -\hat{l}$	
EVV	0.012	1.085	16	980	0.0	1.7	0.051	1.437	55	1000	0.0	28.5
VEV	0.015	1.029	19	950	0.0	1.5	0.072	1.427	74	1000	0.0	27.6
EEV	0.012	0.969	16	930	0.0	0.1	0.074	1.378	80	1000	0.0	3.6
EVE	0.034	1.002	26	928	0.0	1.7	0.908	1.381	505	1000	0.0	26.2
VVE	0.036	0.894	27	874	0.0	0.0	0.872	1.337	487	1000	0.0	1.7
VVV	0.015	0.015	20	15	0.0	0.0	0.039	0.061	44	50	0.0	0.0
EEE	0.010	0.011	14	12	0.0	0.0	0.019	0.019	22	17	0.0	0.0
VVI	0.011	0.010	17	12	0.0	0.0	0.011	0.015	15	15	0.0	0.0
EEI	0.009	0.009	13	11	0.0	0.0	0.009	0.011	11	11	0.0	0.0
VEE	0.012	0.013	17	11	0.0	1.1	0.025	0.013	28	9	0.0	34.5
VII	0.010	0.010	14	11	0.0	0.0	0.006	0.008	8	8	0.0	0.0
VEI	0.011	0.010	16	11	0.0	0.5	0.010	0.008	12	8	0.0	3.6
EVI	0.009	0.009	13	10	0.0	1.0	0.011	0.007	14	6	0.0	7.0
EII	0.007	0.008	10	10	0.0	0.0	0.006	0.007	7	6	0.0	0.0

The columns with header $\hat{l}_{\max } -\hat{l}$ show the average difference between the log-likelihood at convergence and the maximum log-likelihood for that data set. The output is displayed in pairs (FP, MM), which is the result from the FP and MM algorithms

### Real data: parsimony and computational efficiency

Here, we use real data with a twofold aim. Firstly, we show the existence of real-data situations where a parsimonious MLN mixture can provide better performance than its unconstrained version. Secondly, we highlight the gain of the two fitting algorithms here introduced, in terms of computation time, with respect to the one illustrated in Bagnato et al. (2017). We consider the ais (Australian Institute of Sports) data set (Cook and Weisberg 1994) already considered by Bagnato et al. (2017) as an example of a real data situation where the MLN mixture is to be preferred to well-known alternatives in the mixture of elliptical heavy-tailed distributions world; for other applications of this benchmark data set in the model-based clustering literature see, e.g., Azzalini et al. (2016), Dang et al. (2017), Galimberti and Soffritti (2014), and Morris et al. (2019).

**Fig. 3:**
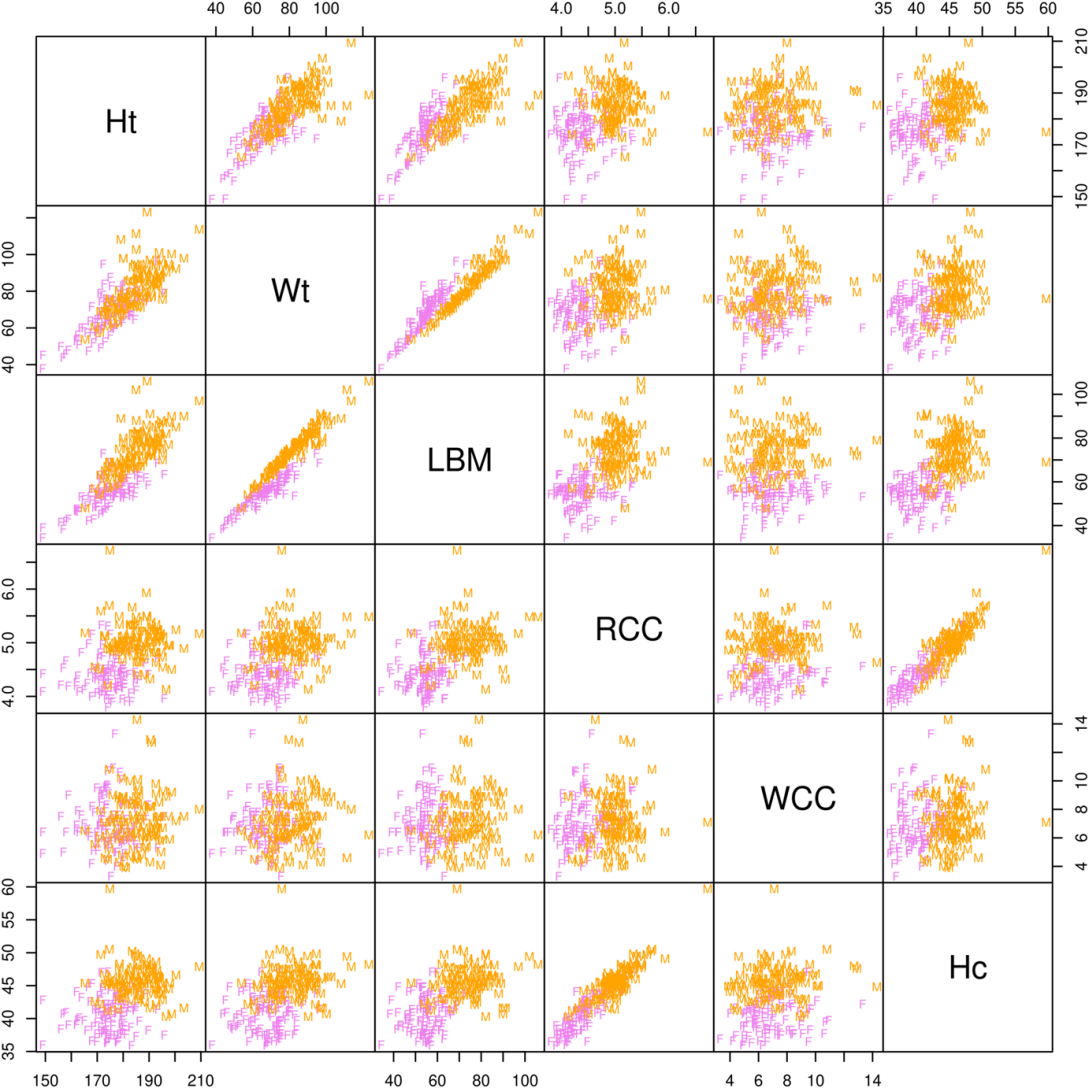
ais data. Scatter plot (M denotes male and F female)

The data set contains measurements on $n=202$ athletes, subdivided in $k=2$ groups (100 female and 102 male), and is available in the R-packages **alr3** (Weisberg 2011) and **sn** (Azzalini 2015). As in Bagnato et al. (2017), we analyze a subset of $d=6$ variables: height in centimeters (Ht), weight in kilograms (Wt), lean body mass (LBM), red cell count (RCC), white cell count (WCC), and Hematocrit (Hc). We show the scatter plot of the data, with labelling based on the gender, in Fig. 3.

On these data, we fit two-component MLN mixtures with four different approaches:

- EM algorithm where a BFGS algorithm, with exact derivatives, is used in each M-step to update all the component parameters;
- EM algorithm where a BFGS algorithm, with numerical derivatives, is used in each M-step to update all the component parameters;
- MM algorithm;
- FP algorithm.

The first two approaches are those considered by Bagnato et al. (2017) for the VVVV model only, while the remaining ones are those proposed herein to fit all the 28 parsimonious models.

For the VVVV model only, Table 3 compares the first two fitting algorithms in terms of Log-likelihood, BIC, ARI, and ratio of the elapsed computational time (over the FP) at convergence. As we can see, they provide the same results, with the exception of the computational time which is much higher (about 690 times higher than the time required by the FP algorithm to fit the same model) when numerical derivatives are used. Anyway, even for the EM with exact derivatives, the computational time required is about 77 times higher than the one required by the FP algorithm.

**Table 3:** Log-likelihood, BIC, ARI, and ratio of the elapsed computational time (over the FP) at convergence of the algorithms adopted by Bagnato et al. (2017) to fit the VVVV MLN mixture

Fitting algorithm	Log-lik	BIC	ARI	Time (over FP)
EM with exact derivatives	$-$2777.461	$-$5857.493	0.811	76.732
EM with numerical derivatives	$-$2777.461	$-$5857.493	0.811	690.049

For all the 28 parsimonious MLN mixtures, Table 4 compares the MM and FP algorithms in terms of log-likelihood values and computational time required. As for the first comparison we note that, for some models, the two algorithms provide different results. A background gray color is used to highlight the algorithm working better. Here we realize that the maximum log-likelihood value provided by the FP algorithm is always greater than, or equal to, the one from to the MM algorithm. This is in line with the results obtained in Sect. 7.1. In terms of computational times, instead, the competition is more heated; however, 15 out of 28 times, the FP algorithm is faster in providing the final solution, and this further corroborates the results in Sect. 7.1. Because of the results on the log-likelihood, Table 3 also reports BIC and ARI values for the solution from the FP algorithm only. For the sake of interpretation, we also report the ranking, among parsimonious model, induced by these criteria.

**Table 4:**
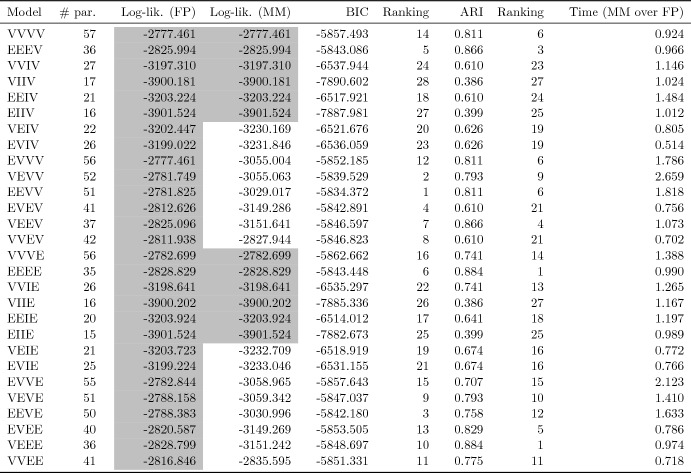
Log-likelihood values and ratio of elapsed computational times (MM over FP) at convergence of the FP and MM algorithms for all the parsimonious MLN mixtures

A background gray color for the best log-likelihood values separately for each model. For the models estimated by the FP algorithm, BIC and ARI values are reported along with the corresponding rankings

The first aspect we note comparing Table 3 with Table 4 is that, for the VVVV model, all the competing fitting algorithms provide the same results, but the MM and FP algorithms, which behave similarly in terms of computational time (ratio of 1.286), obtain these results in a much more fast way. As for the parsimony, the model selected by the BIC (EEVV) provides exactly the same partition of the data given by the VVVV model, but this partition in now obtained via a simpler model with a gain both in terms of estimated parameters (6 parameters less) and interpretation: thanks to the selected model, we realize the two inferred groups are the same in terms volume and shape, but differ in terms of orientation (take a look at the scatterplot between Wt and LBM in Fig. 3) and different excess kurtosis. Finally, by looking at the ARI values in Table 4, a better classification of the data could be obtained by considering the still more parsimonious VEEE and EEEE models, which make the ARI to increase to 0.884.

To find out more about the EEVV model selected by the BIC, we used the parametric bootstrap technique with 10000 bootstrapped samples. The estimates of the parameters along with standard errors (in round brackets) computed by using a parametric bootstrap technique with 10000 bootstrapped samples are showed in Table G.8 shown in Section G of the supplementary material. To tackle the well-known label switching issue in each bootstrap replication, we assigned the labels by minimizing the distance between the estimated and true means, as in Stephens (2000), Farcomeni and Punzo (2020), Gallaugher et al. (2022), and Tomarchio et al. (2022), just to cite a few. The excess kurtosis estimates highlight a further interesting aspect; while one group needs a heavy-tailed distribution to be described in a good way (with a model-based estimated excess kurtosis of 17.083), the other one is well-fitted by a classical normal distribution.

## Investigating some aspects of the MLN mixture

We further investigate the properties of the MLN mixture model when using only the FP method of estimation (Browne et al. 2023). We consider three simulation studies to quantify the sampling variability of the estimation procedure (given in Section E.1 of the supplementary material), the ability of the BIC to pick the correct model (Sect. 8.1), and the effect when data is generated from the *t* or the generalized hyperbolic distributions (in Sect. 8.2).

For these simulations we vary the number of components (*k*), the number of variables (*d*), the distance between the components $(\delta )$, the covariance structure (governed by $\rho$ and $\lambda$, with $\rho \in [0,1)$ and $\lambda >0$), and the sample size (*n*). We fix the $\beta$ parameter to its maximum value, which depends on *d*, in all the simulations. We provide the model parameters, $\varvec{\theta }_k\left( \delta , \lambda , \rho , d\right)$, in a compact form below; for an expanded form, see Section E of the supplementary material. The covariance or scale structure is defined by

15 $$\begin{aligned} \varvec{\Xi }_d\left( \rho \right) = \left( 1-\rho \right) \varvec{I}_d + \rho \varvec{J}_d, \end{aligned}$$

where $\varvec{J}_d$ denotes a $d\times d$ matrix of ones. The two-component setup is

16 $$\begin{aligned}{} & {} \varvec{\theta }_2\left( \delta , \lambda , \rho , d\right) \nonumber \\{} & {} \quad = \left\{ \pi _j = \frac{1}{2}, \; \varvec{\mu }_j = (3-2j)\delta \textbf{1}_d, \; \varvec{\Sigma }_j = \lambda ^{ 3 -2 j } \; \varvec{\Xi }_d(\rho ), \; \beta _j = \frac{4d(d + 2)}{(d + 4)} \right\} , \end{aligned}$$

**Table 5:** Connection between the covariance model structure and the parameters $\lambda$ and $\rho$

Parameters	EIIE	VIIE	EEEE	VEEE
$(\lambda ,\rho )$	(1, 0)	(2, 0)	(1, 0.5)	(2, 0.5)

where $j=1,2$. The three-component setup is

17 $$\begin{aligned}{} & {} \varvec{\theta }_3\left( \delta , \lambda , \rho , d\right) \nonumber \\{} & {} \quad = \left\{ \pi _j = \frac{1}{3}, \; \varvec{\mu }_j = \left( j -2 \right) \delta \textbf{1}_d, \; \varvec{\Sigma }_j = \lambda ^{ (j-2) } \varvec{\Xi }_d\left( \rho \right) , \; \beta _j = \frac{4d(d + 2)}{(d + 4)} \right\} , \end{aligned}$$

where $j=1,2,3$. The four-component setup is

18 $$\begin{aligned}{} & {} \varvec{\theta }_4\left( \delta , \lambda , \rho , d\right) \nonumber \\{} & {} \quad = \left\{ \pi _j = \frac{1}{4}, \; \varvec{\mu }_j = \delta \left[ \begin{array}{c} (-1)^{j} \textbf{1}_d \\ (-1)^{ \left\lfloor \frac{j+1}{2}\right\rfloor } \textbf{1}_d \end{array} \right] \; \varvec{\Sigma }_j = \lambda ^{ \left\lfloor j/2 -1 \right\rfloor } \varvec{\Xi }_d (\rho ), \; \beta _j = \frac{4d(d + 2)}{(d + 4)} \right\} , \end{aligned}$$

where $j=1,2,3,4$ and $\lfloor \cdot \rfloor$ is the floor function, i.e. $\lfloor 1.6 \rfloor = 1$ and $\lfloor -0.6 \rfloor = -1$.

This setup allows to use $\lambda$ and $\rho$ to move through the eigendecomposed or covariance model space. The particular parameter values are given within the forthcoming subsections but of special interest are models given in Table 5. The table shows the connection between some of the covariance models and the parameters.

**Fig. 4:**
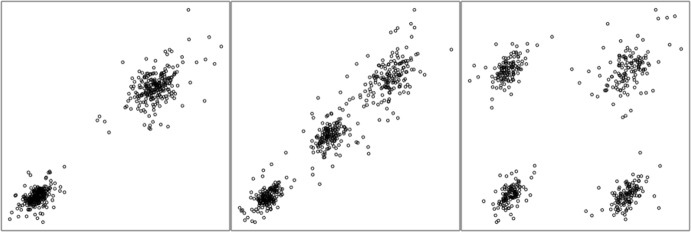
Examples of scatterplots, with $n=500$ observations, from a data generating MLN mixture model with $\varvec{\theta }_k\left( \delta =5, \lambda =2, \rho =0.5, d=2\right)$, with $k=2$ (left), $k=3$ (middle), and $k=4$ (right)

Figure 4 shows three examples of 500 observations generated from $\varvec{\theta }_k\left( \delta =5, \lambda =2, \rho =0.5, d=2\right)$ when varying $k\in \{2,3,4\}$. This is an example of the VEEE model since $\lambda =2$ and $\rho =0.5$.

### Model selection

In this subsection, we investigate how well the BIC picks the correct number of components while varying the distance between clusters ($\delta$), the dimension (*d*), and the sample size (*n*). Three simulation studies were performed to investigate properties of using the BIC: (1) to select the model while fixing *k*, (2) to select *k* while fixing the model, and (3) to jointly select *k* and the model. The parameters explored were generally the same for each of the three studies but some care was needed when setting $\delta$.

First, we examined the ability of the BIC to pick the model while fixing *k*. We considered 72 scenarios arising from the combination of the factors $n\in \{50, 100 \}$, $\rho \in \{0, 0.5\}$, $\lambda \in \{1,2\}$, $d\in \{2,8,16\}$, $k\in \{2,3,4\}$, and $\delta = 3$. For each scenario, 100 data sets were generated and all the models were fitted while using the correct *k*; then, the BIC was used to select the model. Table 6 gives the number of times each model (on the row) was selected by varying the model type, described by the pair $\left( \lambda , \rho \right)$, and the sample size. The values we provide are aggregated over *k* and *d*.

**Table 6:** Number of times each parsimonious model was selected by the BIC

	Data generated from model with $\left( \lambda , \rho \right)$							
	EIIE (1, 0)		VIIE (2, 0)		EEEE (1, 0.5)		VEEE (2, 0.5)	
*n*	50	100	50	100	50	100	50	100
EIIE	816	859	86	37	137	10	28	5
VIIE	41	20	720	830	72	1	192	6
EEEE	3	2	6	2	371	569	93	47
VEEE	1		4	1	27	12	254	517
VVEE	2		2		36	61	140	231
EVEE	2		3		125	200	26	6
VVEV					8	6	42	62
VIIV			37	10	6		34	
EVEV	1				44	24	4	1
EEEV					44	3	8	2
VEEV					2		40	13
EEVE	3		2	2	16	14	11	
EEIE	18	16	8	3			2	
VEVE			4	2	5		19	9
VEIE	1		11	6	1		2	
EVIE	6	1	6	2			1	
EIIV	6	1	4		5			
VVIE			4	2				
EVVE			1		1		2	
VVVE			1				1	1
EVVV		1	1					
VEIV				1				
VVIV				1				
EEVV							1	
VVVV				1				
EEIV								
EVIV								
VEVV								
Total	900	900	900	900	900	900	900	900

Each column represents a summary of 900 data sets generated from a MLN mixture model with $\varvec{\theta }_k\left( \delta , \lambda , \rho , d\right)$ using $k\in \{2,3,4\}$, $d\in \{2,8,16\}$, and $\delta =3$

Second, we examined the ability of the BIC to pick *k* while fixing the model type. We considered 72 scenarios arising from the combination of the factors $n\in \{ 100, 200, 300 \}$, $\rho \in \{0, 0.5\}$, $\lambda \in \{1,2\}$, $d\in \{2,16\}$, and $k\in \{2,3,4\}$. The parameter $\delta$ was chosen so that when *n* varied across its levels, $\{100, 200, 300\}$, the frequency of the number of components selected, say $\tilde{k}$, would be not a degenerate distribution (see Table 7), i.e., if $k=2$ and $\tilde{k}=2$ across *n* would be not illustrate the effect of increasing the sample size. For each scenario, 100 data sets were generated. For each of them, we fitted the MLN mixture model for $k\in \{1,\ldots ,8\}$ while using the correct model; then, the BIC was used to select *k*. Table 7 gives the number of times each value of *k* was selected by the BIC, for each scenario, when $d=2$. For each model, as we increase *n*, the BIC tends to increase the frequency of selecting the correct model. Moving from EIIE to VIIE, or from EEE to VEEE, tends to increases the frequency of $\tilde{k}=k$. When $k=4$, there is more variety in the selected number of components. For the results related to the case $d=16$, see Table E.6 in the supplementary material.

**Table 7:** Number of times each value of *k* is selected by the BIC when fixing the model structure and varying the number of components

Data generated from $\varvec{\theta }_k\left( \delta , \lambda , \rho , d=2\right)$						Selected *k* ($\tilde{k}$)							
*k*	Model	$\rho$	$\lambda$	$\delta$	*n*	1	2	3	4	5	6	7	8
2	EIIE	0.0	1	0.50	100	70	30						
	EIIE	0.0	1	0.50	200	23	77						
	EIIE	0.0	1	0.50	300	6	94						
	EEEE	0.5	1	0.75	100	81	19						
	EEEE	0.5	1	0.75	200	54	46						
	EEEE	0.5	1	0.75	300	34	66						
	VIIE	0.0	2	0.50	100	13	86	1					
	VIIE	0.0	2	0.50	200	1	97	2					
	VIIE	0.0	2	0.50	300		99					1	
	VEEE	0.5	2	0.75	100	17	82	1					
	VEEE	0.5	2	0.75	200		98	1					1
	VEEE	0.5	2	0.75	300		100						
3	EIIE	0.0	1	1.50	100		35	64	1				
	EIIE	0.0	1	1.50	200		6	91	3				
	EIIE	0.0	1	1.50	300		2	96	2				
	EEEE	0.5	1	2.00	100	23	41	36					
	EEEE	0.5	1	2.00	200		15	85					
	EEEE	0.5	1	2.00	300		2	98					
	VIIE	0.0	2	1.50	100		50	43	6			1	
	VIIE	0.0	2	1.50	200		12	85	3				
	VIIE	0.0	2	1.50	300		5	93	2				
	VEEE	0.5	2	2.00	100	5	73	21	1				
	VEEE	0.5	2	2.00	200		37	63					
	VEEE	0.5	2	2.00	300		16	82	1	1			
4	EIIE	0.0	1	1.25	100	69		2	24	5			
	EIIE	0.0	1	1.25	200	3			94	3			
	EIIE	0.0	1	1.25	300				99	1			
	EEEE	0.5	1	1.25	100	20	33	1	40	6			
	EEEE	0.5	1	1.25	200		1		95	4			
	EEEE	0.5	1	1.25	300				100				
	VIIE	0.0	2	1.25	100	39	25	17	18	1			
	VIIE	0.0	2	1.25	200	7	12	16	62	3			
	VIIE	0.0	2	1.25	300			2	95	3			
	VEEE	0.5	2	1.25	100	18	20	27	33	1		1	
	VEEE	0.5	2	1.25	200		1	7	90	1	1		
	VEEE	0.5	2	1.25	300			2	96	2			

Each row refers to 100 data sets generated from a MLN mixture with $\varvec{\theta }_k\left( \delta , \lambda , \rho , d = 2\right)$

Third, we examined the ability of the BIC to pick both the model and *k*. As for the previous case, we considered 72 scenarios arising from the combination of the factors $n\in \{ 100, 200, 300 \}$, $\rho \in \{0, 0.5\}$, $\lambda \in \{1,2\}$, $d\in \{2,16\}$, and $k\in \{2,3,4\}$. Again, the parameter $\delta$ is chosen so that when *n* varied across the values $\{ 100, 200, 300 \}$, the frequency of $\tilde{k}$ would not be a degenerate distribution (see Table 8), i.e., $\delta$ was chosen so that $\tilde{k}\ne k$ for each replication with $n \in \{ 100, 200, 300 \}$. For each scenario, 100 data sets were generated. For each of them, we found the pair of model and *k* selected by the BIC when the search space was $k\in \{1,\ldots ,8\}$ and all the possible model structures. Table 8 gives the number of times each pair $\left( \text {model},k\right)$ was selected by the BIC, for each scenario, when $d=2$. The last two columns give the frequency of picking the number of components and the correct model (second last column) and picking the number of components and one of models in the subset shown in third column group of Table 8. This means that, by definition, the values on the second last column are lower than, or equal to, the values on the last column. For each combination, there is an improvement in the model and component selection when increasing *n* both marginally and jointly. The case $k=3$ has the mostly variety in the chosen model and $k=4$ has the mostly variety in $\tilde{k}$. For the results when $d=16$, see Table E.7 in the supplementary material.

**Table 8:** Counts of the BIC choices, in the case $d=2$, when the search is over $k=1,\ldots ,8$ and all or some of the parsimonious structures

Data generated from $\varvec{\theta }_k\left( \delta , \lambda , \rho , d=2\right)$						Selected *k* ($\tilde{k}$)					Selected fitted model					When $k = \tilde{k}$ and	
*k*	Model	$\lambda$	$\rho$	$\delta$	*n*	1	2	3	4	$\ge 5$	EIIE	VIIE	VEEE	EEEE	VVEE	Model	Subset
2	EIIE	1	0.0	0.5	100	70	29			1	73	5	4	10		22	27
2	EIIE	1	0.0	0.5	200	48	52				76	2	6	9		50	52
2	EIIE	1	0.0	0.5	300	28	71		1		66	6	6	9	3	64	71
2	EEEE	1	0.5	0.8	100	75	22	3			12		22	21	6	8	20
2	EEEE	1	0.5	0.8	200	41	57			2			55	13	4	48	51
2	EEEE	1	0.5	0.8	300	23	77						79	6	1	75	75
2	VIIE	2	0.0	0.5	100	33	60	6		1	43	37	2	3		35	48
2	VIIE	2	0.0	0.5	200	21	67	9	3		21	59	4	2		55	57
2	VIIE	2	0.0	0.5	300	13	68	16	2	1	10	65	1	2	2	58	58
2	VEEE	2	0.5	0.8	100	17	77	5	1		4	9	9	6	54	54	70
2	VEEE	2	0.5	0.8	200		98			2				2	91	90	92
2	VEEE	2	0.5	0.8	300		98			2				2	92	92	94
3	EIIE	1	0.0	1.5	100	18	50	30	2		44	7	13	8	2	24	24
3	EIIE	1	0.0	1.5	200	3	24	70	2	1	69	1	16	3		65	65
3	EIIE	1	0.0	1.5	300		12	86	1	1	84	2	11			83	85
3	EEEE	1	0.5	2.0	100	23	40	35	1	1	10		51	7	10	23	33
3	EEEE	1	0.5	2.0	200	1	22	74	1	2	1		90		6	73	74
3	EEEE	1	0.5	2.0	300		6	92	1	1			92	1	2	87	89
3	VIIE	2	0.0	1.5	100	5	70	23	2		8	56	1	5	13	10	17
3	VIIE	2	0.0	1.5	200		36	62	1	1	4	65		3	15	51	57
3	VIIE	2	0.0	1.5	300		8	91		1		89			4	87	87
3	VEEE	2	0.5	2.0	100	8	70	17	5		7	2	7	18	51	7	14
3	VEEE	2	0.5	2.0	200		52	45	1	2		1	4	21	68	38	44
3	VEEE	2	0.5	2.0	300		24	72	4					11	86	71	71
4	EIIE	1	0.0	1.2	100	37	39	1	22	1	60	8	5			21	22
4	EIIE	1	0.0	1.2	200	4	6	2	88		91					87	87
4	EIIE	1	0.0	1.2	300			1	99		96					96	96
4	EEEE	1	0.5	1.2	100	16	26	5	51	2	31	7	47		3	34	50
4	EEEE	1	0.5	1.2	200	2	3	2	88	5	10	2	84			79	86
4	EEEE	1	0.5	1.2	300				98	2			96		1	95	96
4	VIIE	2	0.0	1.2	100	33	32	23	9	3	42	31		3	1	3	7
4	VIIE	2	0.0	1.2	200	3	9	15	69	4	32	55				39	66
4	VIIE	2	0.0	1.2	300		1	4	93	2	16	82				77	92
4	VEEE	2	0.5	1.2	100	20	37	17	23	3	29	22	20		7	3	21
4	VEEE	2	0.5	1.2	200		5	12	76	7	9	8	29		47	43	75
4	VEEE	2	0.5	1.2	300			4	93	3	2		24		70	67	92

There are four blocks of columns. In the first one, there are the parameters/quantities used to generated the 100 data sets. From the second block onwards, there is the number of times: each value of *k* is picked (2nd block), each model is selected (3rd block), and each pair $\left( \text {model},k\right)$ is selected (4nd block), where “model” belongs to the whole family, in the 2nd last column, and to the subset of models of the 3rd block in the last column

### Data generated from other distributions

In this subsection, we examine the effect on the fitted MLN mixture when the data is generated from another mixture model which exhibits component-wise heavy tails and/or skewness. We consider generating data from finite mixtures of multivariate generalized hyperbolic (GHD) (Browne and McNicholas 2015; Barndorff-Nielsen and Halgreen 1977) and *t* (Peel and McLachlan 2000; Andrews and McNicholas 2012) distributions. We consider a parameter setup similar to the one given in (16) for the two-component MLN mixture, but we replace $\beta$ with the corresponding parameter(s). In particular, for the *t* mixture we have

19 $$\begin{aligned}{} & {} \varvec{\theta }_t \left( \nu \right) \nonumber \\{} & {} \quad = \left\{ \pi _1 = \pi _2 = \frac{1}{2}, \; (\varvec{\mu }_1, \varvec{\mu }_2) = (-3 \textbf{1}_d, +3 \textbf{1}_d), \; \varvec{\Sigma }_j = \varvec{I}_d, \; \nu _1 = \nu _2 = \nu \right\} , \end{aligned}$$

where the degrees of freedom $\nu$ affects the tails, while for the GHD mixture we have

20 $$\begin{aligned}{} & {} \varvec{\theta }_{\text {GHD}} \left( \lambda , \alpha \right) \nonumber \\{} & {} \quad = \left\{ \pi _j = \frac{1}{2}, \; (\varvec{\mu }_1, \varvec{\mu }_2) = (-3 \textbf{1}_d, +3 \textbf{1}_d), \; \varvec{\Sigma }_j = \varvec{I}_d, \; \omega _j=1, \lambda _j = \lambda , (\varvec{\alpha }_1, \varvec{\alpha }_2) = \alpha ( - \varvec{1}_d, \varvec{0}_d) \right\} , \end{aligned}$$

where $\lambda$ affects the tails and $\alpha$ affects the skewness for one of the components.

**Fig. 5:**
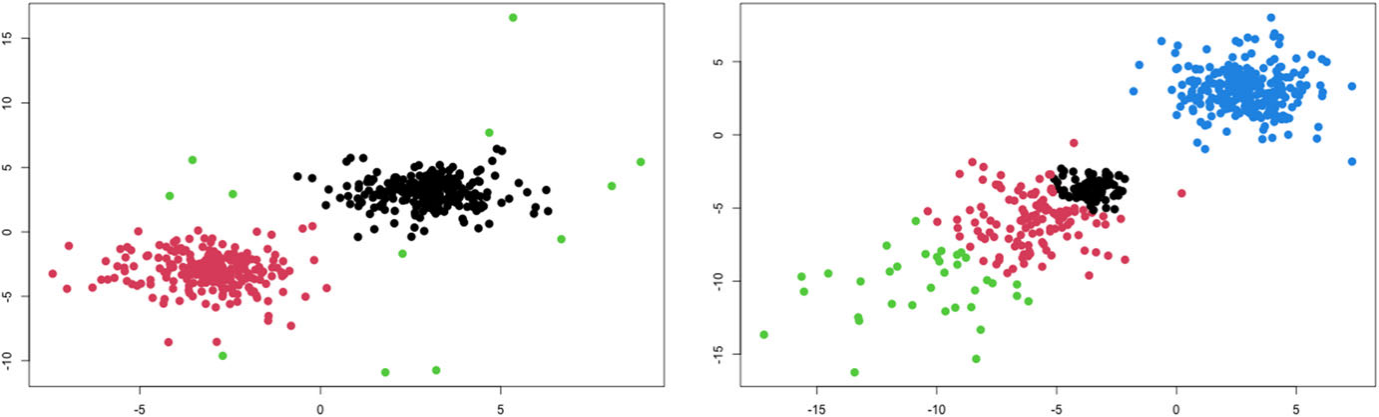
Examples of MLN mixtures fitted to data generated from a 2-component *t* mixture with $\nu =4$ (left panel), and from a 2-component GHD mixture with $(\lambda , \omega , \varvec{\alpha }) = (1,1,-\varvec{1})$ (right panel). The BIC selected the VIIE model with $k=3$, in the left panel, and with $k=4$ in the right panel

Figure 5 shows two examples of the MLN mixture model fitted to generated data. The data in the left panel are generated from a two-component *t* mixture with $\varvec{\theta }_t(4)$. The BIC selected the VIIE MLN mixture model with $k=3$ componnets. Two components match the location of the two multivariate-*t* to the centers, and then the third component is used to model the observations that deviate from normality. The data in the right panel are generated from a GHD mixture with $\varvec{\theta }_{\text {GHD}}(1,-1)$; they display skewness in the bottom-left component. In this case, the BIC still selected the VIIE MLN mixture model, but now with $k=4$ components. One component is used to model the group on the top-right with no skewness, but three components are needed to model the skewness of the other group. In both examples, the ARI between fitted and generated labels was 0.921 (*t* mixture) and 0.729 (GHD mixture).

To investigate the effect of a misspecified model, we generate samples of size $n=500$ from either the *t* or GHD mixture and we fit the MLN mixture with $k=1,\ldots , 8$ and all the 28 possible parsimonious structures. The BIC is used to pick *k* and the model; then, we calculate the ARI between the fitted and generated labels. For the *t*-mixture we use $\nu = \{2, 10\}$ and $d=\{2,8\}$ while for the GHD mixture we use $(\lambda , \alpha )=\left\{ (1, 0), (1,-1), (-1,0), (-1,-1) \right\}$. For both mixtures, we consider $d=\{2,8\}$ and, for each combination of parameters, we generate 100 data sets.

Table 9 shows the frequency of the number of components and the model picked by the BIC when fitting the MLN mixture to data generated from two-component *t* or GHD mixtures. We display all possible $k=1,\ldots ,8$ whereas we only display the four selected models with the highest frequency. The last two columns shows the mean and standard deviations for the ARI. This indicates, on average, that the MLN mixture does will at the group structure even thought the BIC does not pick the correct number of components; e.g., when data are generated from a GHD-mixture with $\alpha =-1$, the BIC picks $k=2$ three times from the 400 replications.

**Table 9:** The frequency of the number of components (*k*) and model picked by the BIC when fitting the MLN mixture to data generated from two-component *t* or GHD mixtures

$\varvec{\theta }_t \left( \nu \right)$	*d*	Selected *k* ($\tilde{k}$)								Selected model				ARI	
		1	2	3	4	5	6	7	8	EIIE	VIIE	VIIV	VVEE	Avg	SD
4	2		41	48	11					88	11	1		0.970	0.019
4	8		4	29	17	19	15	9	7	77	19	4		0.967	0.047
8	2		83	15	2					98	2			0.993	0.007
8	8		74	23	3					92	7			0.999	0.002
$$\varvec{\theta }_{\text {GHD}} (\;\;\lambda , \;\; \alpha )$$															
$(\;\;1, \;\;0)$	2		88	9	3					94	4			0.957	0.017
$(\;\; 1, \;\;0)$	8		71	25	4					75	24	1		0.985	0.050
$( -1, \;\; 0)$	2		74	19	6	1				94	6			0.994	0.015
$( -1, \;\; 0)$	8		33	31	16	20				88	10	1		0.994	0.010
$( \;\;1, -1)$	2		2	46	34	10	5	3		13	36	15	22	0.753	0.060
$( \;\; 1, -1)$	8			27	48	20	4	1			16	40	16	0.691	0.078
$(-1, -1)$	2		1	51	29	16	2	1		33	47	9	2	0.863	0.071
$( -1, -1)$	8			61	19	6	4	4	6	22	33	40		0.825	0.059

## Conclusions

In this paper, we have improved the mixture of multivariate leptokurtic-normal distributions from two points of view. Firstly, we have defined parsimonious variants of the model by putting convenient constraints on the component covariance matrices and excess kurtoses. The use of parsimonious models can lead to more precise parameter estimates and data classifications (Tomarchio et al. 2022). These better performances occur despite the reduction in the number of estimated parameters, allowing for a more parsimonious data modelization. Furthermore, as well documented in the literature (Flury 1988; Greselin et al. 2011; Greselin and Punzo 2013), it should be remarked that parsimonious approaches can disclose richer information on the underlying data structure than the classical unconstrained method. Secondly, we have made maximum likelihood estimation more computationally efficient by introducing two algorithms based on the majorization-minimization algorithm and the fixed point approximation. We highlighted the advantages of our methodological and computational proposals using simulated and real data.

## Supplementary Information

Below is the link to the electronic supplementary material.

Supplementary file 1 (pdf 1497 KB)
