# Supplementary material for: Parsimony and parameter estimation for mixtures of multivariate leptokurtic-normal distributions
Source: Adv Data Anal Classif. 2023 Sep 27;18(3):597–625. doi: 10.1007/s11634-023-00558-2 (PMC11411007; doi:10.1007/s11634-023-00558-2)
Supplement: Supplementary file 1 — (pdf 1497 KB) [file 11634_2023_558_MOESM1_ESM.pdf]

Online Supplementary Material for the paper Parsimony and parameter estimation for mixtures of multivariate leptokurtic-normal distributions

Contents

A Summary of notation and function definitions **2**

B MM algorithm for updating $\boldsymbol{\mu}_j$ and $\boldsymbol{\Sigma}_j$ **4**

B.1 VVV - $\boldsymbol{\Sigma}_j$ 5

B.2 EEE - $\boldsymbol{\Sigma}_j = \boldsymbol{\Sigma}$ 6

B.3 VII - $\boldsymbol{\Sigma}_j = \lambda_j \boldsymbol{I}_d$ 6

B.4 EII - $\boldsymbol{\Sigma}_j = \lambda \boldsymbol{I}_d$ 7

B.5 VVI - $\boldsymbol{\Sigma}_j = \lambda_j \boldsymbol{\Psi}_j = \lambda_j \operatorname{diag}\left(\boldsymbol{\psi}_j\right)$ 7

B.6 EEI - $\boldsymbol{\Sigma}_j = \lambda \boldsymbol{\Psi} = \lambda \operatorname{diag}\left(\boldsymbol{\psi}\right)$ 7

B.7 VEI - $\boldsymbol{\Sigma}_j = \lambda_j \boldsymbol{\Psi} = \lambda_j \operatorname{diag}\left(\boldsymbol{\psi}\right)$ 7

B.8 EVI - $\boldsymbol{\Sigma}_j = \lambda \boldsymbol{\Psi}_j = \lambda \operatorname{diag}\left(\boldsymbol{\psi}_j\right)$ 9

B.9 EEV - $\boldsymbol{\Sigma}_j = \lambda \boldsymbol{\Gamma}_j \boldsymbol{\Psi} \boldsymbol{\Gamma}_j^\top = \lambda \boldsymbol{\Gamma}_j \operatorname{diag}\left(\boldsymbol{\psi}\right) \boldsymbol{\Gamma}_j^\top$ 9

B.10 VVE - $\boldsymbol{\Sigma}_j = \lambda_j \boldsymbol{\Gamma} \boldsymbol{\Psi}_j \boldsymbol{\Gamma}^\top = \lambda_j \boldsymbol{\Gamma} \operatorname{diag}\left(\boldsymbol{\psi}_j\right) \boldsymbol{\Gamma}^\top$ 10

B.11 EVV - $\boldsymbol{\Sigma}_j = \lambda \boldsymbol{\Gamma}_j \boldsymbol{\Psi}_j \boldsymbol{\Gamma}_j^\top = \lambda \boldsymbol{\Gamma}_j \operatorname{diag}\left(\boldsymbol{\psi}_j\right) \boldsymbol{\Gamma}_j^\top$ 11

B.12 VEV - $\boldsymbol{\Sigma}_j = \lambda_j \boldsymbol{\Gamma}_j \boldsymbol{\Psi} \boldsymbol{\Gamma}_j^\top = \lambda_j \boldsymbol{\Gamma}_j \operatorname{diag}\left(\boldsymbol{\psi}\right) \boldsymbol{\Gamma}_j^\top$ 11

B.13 EVE - $\boldsymbol{\Sigma}_j = \lambda \boldsymbol{\Gamma} \boldsymbol{\Psi}_j \boldsymbol{\Gamma}^\top = \lambda \boldsymbol{\Gamma} \operatorname{diag}\left(\boldsymbol{\psi}_j\right) \boldsymbol{\Gamma}^\top$ 12

B.14 VEE - $\boldsymbol{\Sigma}_j = \lambda_j \boldsymbol{\Gamma}\boldsymbol{\Psi}\boldsymbol{\Gamma}^\top = \lambda_j \boldsymbol{\Gamma} \operatorname{diag}(\boldsymbol{\psi}) \boldsymbol{\Gamma}^\top$ 12

C Fixed point iteration with weighted average **13**

D Simulation study: comparing MM and FP algorithms **18**

E Simulation study: Investigating some aspects of the MLN mixture **20**

E.1 Asymptotic properties of the ML estimators 21

E.2 Choosing the number of components 25

F Simulation study: Computational time **32**

A Summary of notation and function definitions

- The function

$$g(r) = \frac{r^2 - 2(d+2)r + d(d+2)}{8d(d+2)},$$

defined in Equation (2) of the paper, adjusts the normal density making it leptokurtic.

- The function $u(r)$, defined in Equation (4) of the paper, is the log-density as a function of the squared Mahalanobis distance. It's first derivative

$$u'(r) = \frac{d}{dr} \log f(r) = \frac{\beta g'(r)}{1 + \beta g(r)} - \frac{1}{2}$$

is used in parameter estimation.

- The function

$$h(r) := \frac{\beta g''(r)}{1 + \beta g(r)} \times 4r - \left[\frac{\beta g'(r)}{1 + \beta g(r)}\right]^2 \times 4r + 2\frac{\beta g'(r)}{1 + \beta g(r)},$$

defined in Equation (5) of the paper, is used to find a bound on the Hessian of the log density.

- The constant

$$M = \left|\min\left\{h(r_1), h(r_2), h(r_3), h(r_4)\right\}\right|,$$

defined in Equation (6) of the paper, is the bound on the Hessian of the log-density.

- The function

$$\gamma_i\left(\boldsymbol{\mu}, \boldsymbol{\Sigma}, \beta\right) = \frac{\beta\ g\left[\left(\boldsymbol{x}_i - \boldsymbol{\mu}\right)^\top \boldsymbol{\Sigma}^{-1}\left(\boldsymbol{x}_i - \boldsymbol{\mu}\right)\right]}{1 + \beta\ g\left[\left(\boldsymbol{x}_i - \boldsymbol{\mu}\right)^\top \boldsymbol{\Sigma}^{-1}\left(\boldsymbol{x}_i - \boldsymbol{\mu}\right)\right]}$$

is used to update β_j.

- The two matrices

$$\boldsymbol{A}_j = \sum_{i=1}^{n} \hat{w}_{ij}^{(q)} \left[2u'\left(r_{ij}^{(q)}\right) + M_j\right] \left(\boldsymbol{x}_i - \boldsymbol{\mu}_j^{(q+1)}\right) \left(\hat{\boldsymbol{z}}_{ij}^{(q)}\right)^\top$$

and

$$\boldsymbol{B}_j = M_j \sum_{i=1}^{n} \hat{w}_{ij}^{(q)} \left(\boldsymbol{x}_i - \boldsymbol{\mu}_j^{(q+1)}\right) \left(\boldsymbol{x}_i - \boldsymbol{\mu}_j^{(q+1)}\right)^\top$$

are used in the MM updates.

- The matrix function $\boldsymbol{G}_{\mathrm{H}}\left(\boldsymbol{A}, \boldsymbol{B}\right)$ provides the solution to continuous time algebraic Riccati equation using the Schur vectors of the associated Hamiltonian which we represent with the following matrix function

$$\boldsymbol{G}_{\mathrm{H}}\left(\boldsymbol{A}, \boldsymbol{B}\right) = \boldsymbol{V}_{21}\boldsymbol{V}_{11}^{-1},$$

where $\boldsymbol{V}_{11}$ and $\boldsymbol{V}_{21}$ are Schur vectors which form an orthogonal transformation that puts the associated Hamiltonian into a quasi-upper-triangular form, i.e.

$$\begin{pmatrix} \boldsymbol{V}_{11} & \boldsymbol{V}_{12} \\ \boldsymbol{V}_{21} & \boldsymbol{V}_{22} \end{pmatrix}^\top \begin{pmatrix} \boldsymbol{A} & -\boldsymbol{B}_j \\ -\boldsymbol{I}_d & -\boldsymbol{A}_j^\top / n_j \end{pmatrix} \begin{pmatrix} \boldsymbol{V}_{11} & \boldsymbol{V}_{12} \\ \boldsymbol{V}_{21} & \boldsymbol{V}_{22} \end{pmatrix} = \begin{pmatrix} \boldsymbol{S}_{11} & \boldsymbol{S}_{12} \\ \boldsymbol{0}_d & \boldsymbol{S}_{22} \end{pmatrix}.$$

These matrices are arranged so that the real parts of the spectrum of $\boldsymbol{S}_{11}$ are negative and the real parts of the spectrum of $\boldsymbol{S}_{22}$ are positive.

- The function $\boldsymbol{h}(\boldsymbol{\psi}, \boldsymbol{a}, \boldsymbol{b})$ represents Algorithm 1 that will be described in Section B.7.

- $|\boldsymbol{U}|_{ll}$ denotes the lth diagonal element of the matrix $\boldsymbol{U}$.

- As for the diag $(\cdot)$ operator, if the input is a matrix, then it extracts the diagonal elements and put them into a vector, while if the input is a vector, then it creates a diagonal matrix with the vector values along the main diagonal.

- $\boldsymbol{G}_{\text{svd}}[\boldsymbol{F}]$ gives the maximizer to $\text{Tr}(\boldsymbol{F\Gamma})$ and it is given by the following matrix operation

$$\boldsymbol{G}_{\text{svd}}[\boldsymbol{F}] := \boldsymbol{Q}\boldsymbol{P}^{\top}, \tag{A.1}$$

  where $\boldsymbol{F}$ has the singular value decomposition $\boldsymbol{F} = \boldsymbol{PDQ}^{\top}$.

- The function

$$\kappa_i(\boldsymbol{\mu}, \boldsymbol{\Sigma}, \beta) = 1 - 2\frac{\beta\, g'\left[(\boldsymbol{x}_i - \boldsymbol{\mu})^{\top} \boldsymbol{\Sigma}^{-1} (\boldsymbol{x}_i - \boldsymbol{\mu})\right]}{1 + \beta\, g\left[(\boldsymbol{x}_i - \boldsymbol{\mu})^{\top} \boldsymbol{\Sigma}^{-1} (\boldsymbol{x}_i - \boldsymbol{\mu})\right]}$$

  is used in the FP algorithm.

B MM algorithm for updating $\boldsymbol{\mu}_j$ and $\boldsymbol{\Sigma}_j$

The minorizer, in terms of the covariance parameters, with updated $\boldsymbol{\mu}_j^{(q+1)}$, is

$$\sum_{j=1}^{k} \left[-\frac{n_j^{(q)}}{2} \log|\boldsymbol{\Sigma}_j| + \text{Tr}\left(\boldsymbol{\Sigma}_j^{-1/2} \boldsymbol{A}_j\right) - \frac{1}{2}\text{Tr}\left(\boldsymbol{\Sigma}_j^{-1} \boldsymbol{B}_j\right) \right], \tag{B.2}$$

where

$$\boldsymbol{A}_j = \sum_{i=1}^{n} w_{ij}^{(q)} \left[2u'\left(r_{ij}^{(q)}\right) + M_j\right] \left(\boldsymbol{x}_i - \boldsymbol{\mu}_j^{(q+1)}\right) \left(\boldsymbol{z}_{ij}^{(q)}\right)^{\top}$$

and

$$\boldsymbol{B}_j = M_j \sum_{i=1}^{n} w_{ij}^{(q)} \left(\boldsymbol{x}_i - \boldsymbol{\mu}_j^{(q+1)}\right) \left(\boldsymbol{x}_i - \boldsymbol{\mu}_j^{(q+1)}\right)^{\top}.$$

Equation (B.2) is the starting point for all the eigen-decomposed models.

B.1 VVV - $\boldsymbol{\Sigma}_j$

To update $\boldsymbol{\Sigma}_j$, we let $\boldsymbol{\Xi}_j = \boldsymbol{\Sigma}_j^{-1/2}$. Then, taking the derivative of (B.2) yields

$$n_j^{(q)} \boldsymbol{I}_d + \boldsymbol{A}_j^\top \boldsymbol{\Xi}_j + \boldsymbol{\Xi}_j \boldsymbol{A}_j - \boldsymbol{\Xi}_j \boldsymbol{B}_j \boldsymbol{\Xi}_j = \boldsymbol{0}.$$

This system is equivalent to the continuous time algebraic Riccati equation (for review, see Wonham, 1968). From Laub (1979, Theorem 5), if $\boldsymbol{I}_d$ and $\boldsymbol{B}_j$ are symmetric and positive definite, then there exists a unique symmetric positive definite solution; in addition, Laub (1979) provides the solution using the Schur vectors of the associated Hamiltonian which we represent with the following matrix function

$$\boldsymbol{\Xi}_j^{(q+1)} = \boldsymbol{G}_{\mathrm{H}} \left(\boldsymbol{A}_j / n_j^{(q)}, \boldsymbol{B}_j / n_j^{(q)} \right) = \boldsymbol{V}_{21} \boldsymbol{V}_{11}^{-1}, \tag{B.3}$$

where $\boldsymbol{V}_{11}$ and $\boldsymbol{V}_{21}$ are Schur vectors which form an orthogonal transformation that puts the associated Hamiltonian into a quasi-upper-triangular form, i.e.

$$\begin{pmatrix} \boldsymbol{V}_{11} & \boldsymbol{V}_{12} \\ \boldsymbol{V}_{21} & \boldsymbol{V}_{22} \end{pmatrix}^\top \begin{pmatrix} \boldsymbol{A}_j / n_j^{(q)} & -\boldsymbol{B}_j / n_j^{(q)} \\ -\boldsymbol{I}_d & -\boldsymbol{A}_j^\top / n_j^{(q)} \end{pmatrix} \begin{pmatrix} \boldsymbol{V}_{11} & \boldsymbol{V}_{12} \\ \boldsymbol{V}_{21} & \boldsymbol{V}_{22} \end{pmatrix} = \begin{pmatrix} \boldsymbol{S}_{11} & \boldsymbol{S}_{12} \\ \boldsymbol{0}_d & \boldsymbol{S}_{22} \end{pmatrix}.$$

These matrices are arranged so that the real parts of the spectrum of $\boldsymbol{S}_{11}$ are negative and the real parts of the spectrum of $\boldsymbol{S}_{22}$ are positive. Calculating the Schur eigenvectors, sorted by the eigenvalues, is implemented in Lapack by Anderson et al. (1999); then the update for $\boldsymbol{\Sigma}_j$ is $\boldsymbol{\Sigma}_j^{(t+1)} = \left[\boldsymbol{\Xi}_j^{(q+1)} \right]^{-2}$.

B.2 EEE - $\boldsymbol{\Sigma}_j = \boldsymbol{\Sigma}$

To update $\boldsymbol{\Sigma}$, we let $\boldsymbol{\Xi} = \boldsymbol{\Sigma}^{-1/2}$. Then, taking the derivative of (B.2) with respect to $\boldsymbol{\Xi}$ yields another continuous time algebraic Riccati equation which has solution given by

$$\boldsymbol{\Xi}^{(q+1)} = \boldsymbol{G}_{\mathrm{H}}\left(\frac{1}{n}\sum_{j=1}^{k}\boldsymbol{B}_j, \frac{1}{n}\sum_{j=1}^{k}\boldsymbol{A}_j\right),$$

and then again we reparameterize to obtain $\boldsymbol{\Sigma}^{(q+1)}$.

B.3 VII - $\boldsymbol{\Sigma}_j = \lambda_j \boldsymbol{I}_d$

Under this parameterization, (B.2) reduces to

$$\sum_{j=1}^{k}\left[-\frac{n_j^{(q)}d}{2}\log\lambda_j + \frac{1}{\sqrt{\lambda_j}}\operatorname{Tr}(\boldsymbol{A}_j) - \frac{1}{2\lambda_j}\operatorname{Tr}(\boldsymbol{B}_j)\right].$$

Letting $\xi_j = \lambda_j^{-1/2}$ and taking the derivative with respect to ξ_j yields the following quadratic equation,

$$n_j^{(q)}d + \xi_j \times \operatorname{Tr}[\boldsymbol{A}_j] - \xi_j^2 \times \operatorname{Tr}[\boldsymbol{B}_j] = 0.$$

An equivalent quadratic equation is $1 + az - bz^2$, which has roots

$$v(a,b) := \frac{a+\sqrt{a^2+4b}}{2b} \qquad \text{and} \qquad \frac{a-\sqrt{a^2+4b}}{2b}. \tag{B.4}$$

Because $b > 0$, the first root, that we define as $v(a,b)$, is positive and the second one is negative. So the update for ξ_j can be defined as

$$\xi_j^{(q+1)} = v\left[\frac{1}{d}\operatorname{Tr}\left(\boldsymbol{A}_j/n_j^{(q)}\right), \frac{1}{d}\operatorname{Tr}\left(\boldsymbol{B}_j/n_j^{(q)}\right)\right], \tag{B.5}$$

and $\lambda_j^{(q+1)} = \left[\xi_j^{(q+1)}\right]^{-2}$.

B.4 EII - $\boldsymbol{\Sigma}_j = \lambda \boldsymbol{I}_d$

By letting $\xi = \lambda^{-1/2}$, and taking the derivative with respect to ξ, yields the update

$$\xi^{(q+1)} = v\left[\operatorname{Tr}\left(\sum_{j=1}^{k} \boldsymbol{A}_j/n\right), \operatorname{Tr}\left(\sum_{j=1}^{k} \boldsymbol{B}_j/n\right)\right], \tag{B.6}$$

with $\lambda^{(q+1)} = \left[\xi^{(q+1)}\right]^{-2}$.

B.5 VVI - $\boldsymbol{\Sigma}_j = \lambda_j \boldsymbol{\Psi}_j = \lambda_j \operatorname{diag}\left(\boldsymbol{\psi}_j\right)$

Let $\boldsymbol{\psi}_j$ be a d-dimensional vector. By letting $\xi_{jl} = \lambda_j \psi_l$, and taking the derivative with respect to ξ_{gj}, yields the following updates

$$\xi_{jl}^{(q+1)} = v\left(\frac{1}{n_j^{(q)}} \left|\boldsymbol{A}_j\right|_{ll}, \frac{1}{n_j^{(q)}} \left|\boldsymbol{B}_j\right|_{ll}\right) \tag{B.7}$$

for each $l = 1, \ldots, d$, $j = 1, \ldots, k$, where $|\boldsymbol{U}|_{ll}$ denotes the lth diagonal element of the matrix $\boldsymbol{U}$. Then, we can reparameterize to obtain updates for λ_j and $\boldsymbol{\psi}_j$.

B.6 EEI - $\boldsymbol{\Sigma}_j = \lambda \boldsymbol{\Psi} = \lambda \operatorname{diag}(\boldsymbol{\psi})$

Let $\boldsymbol{\psi}$ be a d-dimensional vector. By letting $\xi_l = \lambda \psi_l$, and taking the derivative with respect to each ξ_l, yields a quadratic equation whose solution is

$$\xi_l^{(q+1)} = v\left(\frac{1}{n}\sum_{j=1}^{k} \left|\boldsymbol{A}_j\right|_{ll}, \frac{1}{n}\sum_{j=1}^{k} \left|\boldsymbol{B}_j\right|_{ll}\right).$$

B.7 VEI - $\boldsymbol{\Sigma}_j = \lambda_j \boldsymbol{\Psi} = \lambda_j \operatorname{diag}(\boldsymbol{\psi})$

The surrogate function in (B.2) becomes

$$\sum_{j=1}^{k}\left[-\frac{dn_j^{(q)}}{2}\log\lambda_j + \frac{1}{\sqrt{\lambda_j}}\operatorname{Tr}\left(\boldsymbol{\Psi}^{-1/2}\boldsymbol{A}_j\right) - \frac{1}{2\lambda_j}\operatorname{Tr}\left(\boldsymbol{\Psi}^{-1}\boldsymbol{B}_j\right)\right] \qquad \text{subject to } |\boldsymbol{\Psi}| = 1.$$

Conditional on $\boldsymbol{\Psi}$, an update for $\xi_j = \lambda_j^{-1/2}$ is

$$\xi_j^{(q+1)} = v\left[\operatorname{Tr}\left(\boldsymbol{\Psi}^{-1/2}\boldsymbol{A}_j/n_j^{(q)}\right), \operatorname{Tr}\left(\boldsymbol{\Psi}^{-1}\boldsymbol{B}_j/n_j^{(q)}\right)\right],$$

and $\lambda_j^{(q+1)} = \left[\xi_j^{(q+1)}\right]^{-2}$. Then, conditional on $\lambda_j = \lambda_j^{(q+1)}$, an update for $\boldsymbol{\psi}$ can be obtained by a reparamerization to yield a linear constraint. That is, we let $\eta_j = -1/2\log\psi_l$ and obtain

$$\sum_{i=1}^{j} e^{\eta_j}\left(\sum_{j=1}^{k}\frac{|\boldsymbol{A}_j|_{ll}}{\sqrt{\lambda_j^{(q+1)}}}\right) - \frac{1}{2}\sum_{i=1}^{j} e^{2\eta_j}\left(\sum_{j=1}^{k}\frac{|\boldsymbol{B}_j|_{ll}}{\lambda_j^{(q+1)}}\right) \qquad \text{subject to} \sum_{j=1}^{d}\eta_j = 0.$$

The constraint is now linear and we can use the elimination of variables (Nocedal and Wright, 2006, Section 15.3). By letting $\eta_1 = -\sum_{j=2}^{d}\eta_j$, $a_l = \sum_{j=1}^{k}|\boldsymbol{A}_j|_{ll}/\sqrt{\lambda_j^{(q+1)}}$ and $b_l = \sum_{j=1}^{k}|\boldsymbol{B}_j|_{ll}/\lambda_j^{(q+1)}$, we can form the unconstrained problem

$$a_1 e^{-\sum_{l=2}^{d}\eta_j} - \frac{b_1}{2}e^{-2\sum_{l=2}^{d}\eta_l} + \sum_{l=2}^{d}\left(a_l e^{\eta_l} - \frac{b_l}{2}e^{2\eta_l}\right).$$

We then perform Newton-Raphson on this unconstrained problem and reparameterize to update $\boldsymbol{\psi}$. Algorithm 1 defines the function which performs this procedure. Note, one can use the Sherman-

---

Algorithm 1 The function $\boldsymbol{h}(\boldsymbol{\psi}, \boldsymbol{a}, \boldsymbol{b})$ has inputs the vectors $\boldsymbol{\psi} \in \mathbb{R}_+^d$ such that $\prod_{l=1}^{d}\psi_l = 1$ and the vectors $\boldsymbol{a} \in \mathbb{R}^d$ and $\boldsymbol{b} \in \mathbb{R}_+^d$. Note, $\boldsymbol{\eta}$ is of dimension $d-1$.

Obtain $\boldsymbol{\eta}$ by setting $\eta_l = -1/2\log\psi_l$ for $l = 2, \ldots, d$.
Calculate the gradient and Hessian

$$\boldsymbol{g} = \left(a_2 e^{\eta_2} - b_2 e^{2\eta_2}, \ldots, a_d e^{\eta_d} - b_d e^{2\eta_d}\right) - \left(a_1 e^{-\sum_{l=2}^{d}\eta_l} - b_1 e^{-2\sum_{l=2}^{d}\eta_l}\right)\mathbf{1}_{d-1},$$
$$\boldsymbol{H} = \operatorname{diag}\left(a_2 e^{-\eta_2} - 2b_2 e^{2\eta_2}, \ldots, a_d e^{-\eta_d} - 2b_d e^{2\eta_d}\right) - \left(a_1 e^{-\sum_{l=2}^{d}\eta_l} - 2b_1 e^{-2\sum_{l=2}^{d}\eta_l}\right)\mathbf{1}_{d-1}\mathbf{1}_{d-1}^{\top}.$$

Perform Netwon-Raphson
Obtain $\boldsymbol{\psi}$ by setting $\psi_l = e^{-2\eta_l}$ for $l = 2, \ldots, d$ and $\psi_1 = e^{-2\sum_{l=2}^{d}\eta_l}$.
return $\boldsymbol{\psi}$

Morrison formula to find the inverse of the Hessian. We define the function $\boldsymbol{h}$ to represent algo-

rithm 1 and then the update can be written as

$$\boldsymbol{\psi}^{(q+1)} = \boldsymbol{h}\left(\boldsymbol{\psi}^{(q)}, \sum_{j=1}^{k} \frac{\operatorname{diag}(\boldsymbol{A}_j)}{\sqrt{\lambda_j^{(q+1)}}}, \sum_{j=1}^{k} \frac{\operatorname{diag}(\boldsymbol{B}_j)}{\lambda_j^{(q+1)}}\right).$$

B.8 EVI - $\boldsymbol{\Sigma}_j = \lambda \boldsymbol{\Psi}_j = \lambda \operatorname{diag}(\boldsymbol{\psi}_j)$

Conditional on $\boldsymbol{\Psi}_j$, an update for $\xi = \lambda^{-1/2}$ is

$$\xi^{(q+1)} = v\left[\frac{1}{n}\sum_{j=1}^{k} \operatorname{Tr}\left(\boldsymbol{\Psi}^{-1/2}\boldsymbol{A}_j\right), \frac{1}{n}\sum_{j=1}^{k} \operatorname{Tr}\left(\boldsymbol{\Psi}^{-1}\boldsymbol{B}_j\right)\right].$$

The utilize of Algorithm 1 gets the following update

$$\boldsymbol{\psi}_j^{(q+1)} = \boldsymbol{h}\left(\boldsymbol{\psi}_j^{(q)}, \frac{\operatorname{diag}(\boldsymbol{A}_j)}{\sqrt{\lambda^{(q+1)}}}, \frac{\operatorname{diag}(\boldsymbol{B}_j)}{\lambda^{(q+1)}}\right).$$

B.9 EEV - $\boldsymbol{\Sigma}_j = \lambda \boldsymbol{\Gamma}_j \boldsymbol{\Psi} \boldsymbol{\Gamma}_j^\top = \lambda \boldsymbol{\Gamma}_j \operatorname{diag}(\boldsymbol{\psi}) \boldsymbol{\Gamma}_j^\top$

By conditioning on $\boldsymbol{\Gamma}_j$, and letting $\xi_l = \lambda \psi_l$, we can obtain the following update

$$\xi_l^{(q+1)} = v\left(\frac{1}{n_j^{(q)}}\left|\boldsymbol{\Gamma}_j^\top \boldsymbol{A}_j \boldsymbol{\Gamma}_j\right|_{ll}, \frac{1}{n_j^{(q)}}\left|\boldsymbol{\Gamma}_j^\top \boldsymbol{B}_j \boldsymbol{\Gamma}_j\right|_{ll}\right)$$

and reparameterize it to update λ and ψ_l. If we let $\boldsymbol{\Lambda} = \lambda \operatorname{diag}(\boldsymbol{\psi})$, then (B.2) as function of $\boldsymbol{\Gamma}_j$ becomes

$$\operatorname{Tr}\left(\boldsymbol{\Gamma}_j \boldsymbol{\Lambda}^{-1/2} \boldsymbol{\Gamma}_j^\top \boldsymbol{A}_j\right) - \frac{1}{2}\operatorname{Tr}\left(\boldsymbol{\Gamma}_j \boldsymbol{\Lambda}^{-1} \boldsymbol{\Gamma}_j^\top \boldsymbol{B}_j\right) \quad \text{subject to } \boldsymbol{\Gamma}_j^\top \boldsymbol{\Gamma}_j = \boldsymbol{I}_d.$$

An iterative procedure can be obtained using an accelerated line search (Browne and McNicholas, 2014b; Absil et al., 2008), but here we will follow Browne and McNicholas (2014a) and Kiers (2002) and construct a MM algorithm. Then, to update $\boldsymbol{\Gamma}_j$ we view (B.2) as

$$\operatorname{Tr}\left(\boldsymbol{\Gamma}_j \boldsymbol{\Lambda}_j^{-1/2} \boldsymbol{\Gamma}_j^\top \boldsymbol{A}_j\right) - \frac{1}{2}\operatorname{Tr}\left(\boldsymbol{\Gamma}_j \boldsymbol{\Lambda}_j^{-1} \boldsymbol{\Gamma}_j^\top \boldsymbol{B}_j\right) \geq C + \operatorname{Tr}\left[\boldsymbol{F}\left(\boldsymbol{\Gamma}^{(q)}, \boldsymbol{\Lambda}_j, \boldsymbol{A}_j, \boldsymbol{B}_j\right)\boldsymbol{\Gamma}_j\right],$$

with the matrix function, $\boldsymbol{F}$, defined as

$$\boldsymbol{F}(\boldsymbol{\Gamma}, \boldsymbol{\Lambda}, \boldsymbol{A}, \boldsymbol{B}) := \boldsymbol{\Gamma}^\top \left[\frac{1}{\alpha}\boldsymbol{B}^\top + \frac{1}{\sqrt{\alpha}}\left(\boldsymbol{A}^\top + \boldsymbol{A}\right)\right] + \boldsymbol{\Lambda}^{-1/2}\boldsymbol{\Gamma}^\top\left(\boldsymbol{A}^\top + \boldsymbol{A}\right) - \boldsymbol{\Lambda}^{-1}\boldsymbol{\Gamma}^\top\boldsymbol{B}^\top, \tag{B.8}$$

where α is the smallest eigenvalue of $\boldsymbol{\Lambda}$.

According to Cliff (1996), the maximizer to $\mathrm{Tr}(\boldsymbol{F}\boldsymbol{\Gamma})$ is given by following matrix operation

$$\boldsymbol{G}_{\mathrm{svd}}[\boldsymbol{F}] := \boldsymbol{Q}\boldsymbol{P}^\top, \tag{B.9}$$

where $\boldsymbol{F}$ has the following singular value decomposition $\boldsymbol{F} = \boldsymbol{P}\boldsymbol{D}\boldsymbol{Q}^\top$. Applying (B.8) and (B.9), the update can be written as

$$\boldsymbol{\Gamma}_j^{(q+1)} = \boldsymbol{G}_{\mathrm{svd}}\left[\boldsymbol{F}\left(\boldsymbol{\Gamma}^{(q)}, \boldsymbol{\Lambda}_j^{(q+1)}, \boldsymbol{A}_j, \boldsymbol{B}_j\right)\right].$$

B.10 VVE - $\boldsymbol{\Sigma}_j = \lambda_j\boldsymbol{\Gamma}\boldsymbol{\Psi}_j\boldsymbol{\Gamma}^\top = \lambda_j\boldsymbol{\Gamma}\,\mathrm{diag}\left(\boldsymbol{\psi}_j\right)\boldsymbol{\Gamma}^\top$

By conditioning on $\boldsymbol{\Gamma}$, and letting $\xi_{jl} = \lambda_j\psi_{jl}$, we can obtain following update

$$\xi_{jl}^{(q+1)} = v\left(\frac{1}{n_j^{(q)}}\left|\boldsymbol{\Gamma}^\top\boldsymbol{A}_j\boldsymbol{\Gamma}\right|_{ll}, \frac{1}{n_j^{(q)}}\left|\boldsymbol{\Gamma}^\top\boldsymbol{B}_j\boldsymbol{\Gamma}\right|_{ll}\right),$$

and reparameterizing it to update λ_j and ψ_{jl}. If we let $\boldsymbol{\Lambda}_j = \lambda_j\,\mathrm{diag}\left(\boldsymbol{\psi}_j\right)$, then (B.2), as a function of $\boldsymbol{\Gamma}$, becomes

$$\sum_{j=1}^{k}\left[\mathrm{Tr}\left(\boldsymbol{\Gamma}\boldsymbol{\Lambda}_j^{-1/2}\boldsymbol{\Gamma}^\top\boldsymbol{A}_j\right) - \frac{1}{2}\mathrm{Tr}\left(\boldsymbol{\Gamma}\boldsymbol{\Lambda}_j^{-1}\boldsymbol{\Gamma}^\top\boldsymbol{B}_j\right)\right] \quad \text{subject to } \boldsymbol{\Gamma}^\top\boldsymbol{\Gamma} = \boldsymbol{I}_d.$$

Utilizing (B.8) and (B.9), then we have the following update

$$\boldsymbol{\Gamma}^{(q+1)} = \boldsymbol{G}_{\mathrm{svd}}\left[\sum_{j=1}^{k}\boldsymbol{F}\left(\boldsymbol{\Gamma}^{(q)}, \boldsymbol{\Lambda}^{(q+1)}, \boldsymbol{A}_j, \boldsymbol{B}_j\right)\right].$$

B.11 EVV - $\boldsymbol{\Sigma}_j = \lambda \boldsymbol{\Gamma}_j \boldsymbol{\Psi}_j \boldsymbol{\Gamma}_j^\top = \lambda \boldsymbol{\Gamma}_j \operatorname{diag}\left(\boldsymbol{\psi}_j\right) \boldsymbol{\Gamma}_j^\top$

Conditional on $\boldsymbol{\Psi}_j$ and $\boldsymbol{\Gamma}_j$, an update for $\xi = \lambda^{-1/2}$ is

$$\xi^{(q+1)} = v\left[\frac{1}{n_j^{(q)}} \operatorname{Tr}\left(\boldsymbol{\Psi}_j^{-1/2} \boldsymbol{\Gamma}_j^\top \boldsymbol{A}_j \boldsymbol{\Gamma}_j\right), \frac{1}{n_j^{(q)}} \operatorname{Tr}\left(\boldsymbol{\Psi}^{-1} \boldsymbol{\Gamma}_j^\top \boldsymbol{B}_j \boldsymbol{\Gamma}_j\right)\right].$$

Then, we obtain $\lambda^{(q+1)} = \left[\xi^{(q+1)}\right]^{-2}$ and utilize Algorithm 1 to get the following update

$$\boldsymbol{\psi}_j^{(q+1)} = \boldsymbol{h}\left(\boldsymbol{\psi}_j^{(q)}, \frac{\operatorname{diag}\left(\boldsymbol{\Gamma}_j^\top \boldsymbol{A}_j \boldsymbol{\Gamma}_j\right)}{\sqrt{\lambda^{(q+1)}}}, \frac{\operatorname{diag}\left(\boldsymbol{\Gamma}_j^\top \boldsymbol{B}_j \boldsymbol{\Gamma}_j\right)}{\lambda^{(q+1)}}\right).$$

Finally, we construct $\boldsymbol{\Lambda}_j^{(q+1)} = \lambda^{(q+1)} \operatorname{diag}\left(\boldsymbol{\psi}_j^{(q+1)}\right)$ and obtain the update

$$\boldsymbol{\Gamma}_j^{(q+1)} = \boldsymbol{G}_{\text{svd}}\left[\boldsymbol{F}\left(\boldsymbol{\Gamma}_j^{(q)}, \boldsymbol{\Lambda}_j^{(q+1)}, \boldsymbol{A}_j, \boldsymbol{B}_j\right)\right].$$

B.12 VEV - $\boldsymbol{\Sigma}_j = \lambda_j \boldsymbol{\Gamma}_j \boldsymbol{\Psi} \boldsymbol{\Gamma}_j^\top = \lambda_j \boldsymbol{\Gamma}_j \operatorname{diag}\left(\boldsymbol{\psi}\right) \boldsymbol{\Gamma}_j^\top$

Conditional on $\boldsymbol{\Psi}$ and $\boldsymbol{\Gamma}_j$, an update for $\xi_j = \lambda_j^{-1/2}$ is

$$\xi_j^{(q+1)} = v\left[\frac{1}{n_j^{(q)}} \operatorname{Tr}\left(\boldsymbol{\Psi}^{-1/2} \boldsymbol{\Gamma}_j^\top \boldsymbol{A}_j \boldsymbol{\Gamma}_j\right), \frac{1}{n_j^{(q)}} \operatorname{Tr}\left(\boldsymbol{\Psi}^{-1} \boldsymbol{\Gamma}_j^\top \boldsymbol{B}_j \boldsymbol{\Gamma}_j\right)\right].$$

Then, we obtain $\lambda^{(q+1)} = \left[\xi^{(q+1)}\right]^{-2}$ and utilize Algorithm 1 to get the following update

$$\boldsymbol{\psi}^{(q+1)} = \boldsymbol{h}\left(\boldsymbol{\psi}^{(q)}, \sum_{j=1}^{k} \frac{\operatorname{diag}\left(\boldsymbol{\Gamma}_j^\top \boldsymbol{A}_j \boldsymbol{\Gamma}_j\right)}{\sqrt{\lambda^{(q+1)}}}, \sum_{j=1}^{k} \frac{\operatorname{diag}\left(\boldsymbol{\Gamma}_j^\top \boldsymbol{B}_j \boldsymbol{\Gamma}_j\right)}{\lambda^{(q+1)}}\right). \tag{B.10}$$

Finally, we construct $\boldsymbol{\Lambda}_j^{(q+1)} = \lambda_j^{(q+1)} \operatorname{diag}\left(\boldsymbol{\psi}^{(q+1)}\right)$ and obtain the update

$$\boldsymbol{\Gamma}_j^{(q+1)} = \boldsymbol{G}_{\text{svd}}\left[\boldsymbol{F}\left(\boldsymbol{\Gamma}_j^{(q)}, \boldsymbol{\Lambda}_j^{(q+1)}, \boldsymbol{A}_j, \boldsymbol{B}_j\right)\right].$$

B.13 EVE - $\boldsymbol{\Sigma}_j = \lambda \boldsymbol{\Gamma} \boldsymbol{\Psi}_j \boldsymbol{\Gamma}^\top = \lambda \boldsymbol{\Gamma} \operatorname{diag}\left(\boldsymbol{\psi}_j\right) \boldsymbol{\Gamma}^\top$

Conditional on $\boldsymbol{\Psi}_j$ and $\boldsymbol{\Gamma}$, an update for $\xi = \lambda^{-1/2}$ is

$$\xi^{(q+1)} = v \left[\sum_{j=1}^{k} \operatorname{Tr}\left(\boldsymbol{\Psi}_j^{-1/2} \boldsymbol{\Gamma}^\top \boldsymbol{A}_j \boldsymbol{\Gamma} \right), \sum_{j=1}^{k} \operatorname{Tr}\left(\boldsymbol{\Psi}_j^{-1} \boldsymbol{\Gamma}^\top \boldsymbol{B}_j \boldsymbol{\Gamma} \right) \right].$$

Then, we obtain $\lambda^{(q+1)} = \left[\xi^{(q+1)}\right]^{-2}$ and utilize Algorithm 1 to get the following update

$$\boldsymbol{\psi}_j^{(q+1)} = \boldsymbol{h}\left(\boldsymbol{\psi}^{(q)}, \frac{\operatorname{diag}\left(\boldsymbol{\Gamma}^\top \boldsymbol{A}_j \boldsymbol{\Gamma}\right)}{\sqrt{\lambda^{(q+1)}}}, \frac{\operatorname{diag}\left(\boldsymbol{\Gamma}^\top \boldsymbol{B}_j \boldsymbol{\Gamma}\right)}{\lambda^{(q+1)}} \right).$$

Finally, we construct $\boldsymbol{\Lambda}_j^{(q+1)} = \lambda^{(q+1)} \operatorname{diag}\left(\boldsymbol{\psi}_j^{(q+1)}\right)$ and obtain the update

$$\boldsymbol{\Gamma}^{(q+1)} = \boldsymbol{G}_{\text{svd}} \left[\boldsymbol{F}\left(\boldsymbol{\Gamma}^{(q)}, \boldsymbol{\Lambda}_j^{(q+1)}, \boldsymbol{A}_j, \boldsymbol{B}_j \right) \right].$$

B.14 VEE - $\boldsymbol{\Sigma}_j = \lambda_j \boldsymbol{\Gamma} \boldsymbol{\Psi} \boldsymbol{\Gamma}^\top = \lambda_j \boldsymbol{\Gamma} \operatorname{diag}\left(\boldsymbol{\psi}\right) \boldsymbol{\Gamma}^\top$

The solutions for each individual quadratic equation are obtained analogously to (B.5) and (B.6). Conditional on $\boldsymbol{\Psi}$, an update for $\xi_j = \lambda_j^{-1/2}$ is

$$\xi_j^{(q+1)} = v \left[\operatorname{Tr}\left(\boldsymbol{\Psi}^{-1/2} \boldsymbol{\Gamma}^\top \boldsymbol{A}_j \boldsymbol{\Gamma} \right), \operatorname{Tr}\left(\boldsymbol{\Psi}^{-1} \boldsymbol{\Gamma}^\top \boldsymbol{B}_j \boldsymbol{\Gamma} \right) \right]. \tag{B.11}$$

Then, we obtain $\lambda_j^{(q+1)} = \left[\xi_j^{(q+1)}\right]^{-2}$ and utilize Algorithm 1 to get the following update

$$\boldsymbol{\psi}^{(q+1)} = \boldsymbol{h}\left(\boldsymbol{\psi}^{(q)}, \sum_{j=1}^{k} \frac{\operatorname{diag}\left(\boldsymbol{\Gamma}^\top \boldsymbol{A}_j \boldsymbol{\Gamma}\right)}{\sqrt{\lambda_j^{(q+1)}}}, \sum_{j=1}^{k} \frac{\operatorname{diag}\left(\boldsymbol{\Gamma}^\top \boldsymbol{B}_j \boldsymbol{\Gamma}\right)}{\lambda_j^{(q+1)}} \right).$$

Finally, we construct $\boldsymbol{\Lambda}_j^{(q+1)} = \lambda_j^{(q+1)} \operatorname{diag}\left(\boldsymbol{\psi}^{(q+1)}\right)$ and obtain the update

$$\boldsymbol{\Gamma}^{(q+1)} = \boldsymbol{G}_{\text{svd}} \left[\sum_{j=1}^{k} \boldsymbol{F}\left(\boldsymbol{\Gamma}^{(q)}, \boldsymbol{\Lambda}_j^{(q+1)}, \boldsymbol{A}_j, \boldsymbol{B}_j \right) \right].$$

C Fixed point iteration with weighted average

For a single component and having $(\boldsymbol{\mu}, \beta)$ fixed, the update for $\boldsymbol{\Sigma}_j$ is based on the following quantity

$$\boldsymbol{R}^{(q)} = \frac{1}{n} \sum_{i=1}^{n} \kappa_i \left(\boldsymbol{\mu}, \boldsymbol{\Sigma}^{(q)}, \beta \right) (\boldsymbol{x}_i - \boldsymbol{\mu}) (\boldsymbol{x}_i - \boldsymbol{\mu})^\top .$$

and note that κ_i can be written as

$$\kappa_i (\boldsymbol{\mu}, \boldsymbol{\Sigma}, \beta) = \kappa_\beta \left(r = (\boldsymbol{x}_i - \boldsymbol{\mu})^\top \boldsymbol{\Sigma}^{-1} (\boldsymbol{x}_i - \boldsymbol{\mu}) \right), \quad \text{where} \quad \kappa_\beta (r) = 1 - 2 \frac{\beta \; g'(r)}{1 + \beta \; g(r)}.$$

So, the update can be viewed as fixed point iteration

$$\boldsymbol{M} (\boldsymbol{\Sigma}) = \frac{1}{n} \sum_{i=1}^{n} \kappa_\beta \left[(\boldsymbol{x}_i - \boldsymbol{\mu})^\top \boldsymbol{\Sigma}^{-1} (\boldsymbol{x}_i - \boldsymbol{\mu}) \right] (\boldsymbol{x}_i - \boldsymbol{\mu}) (\boldsymbol{x}_i - \boldsymbol{\mu})^\top . \tag{C.12}$$

To take the derivative we apply the vectorization,

$$\text{vec} \boldsymbol{M} (\boldsymbol{\Sigma}) = \frac{1}{n} \sum_{i=1}^{n} \kappa_\beta \left[(\boldsymbol{x}_i - \boldsymbol{\mu})^\top \boldsymbol{\Sigma}^{-1} (\boldsymbol{x}_i - \boldsymbol{\mu}) \right] \text{vec} \left[(\boldsymbol{x}_i - \boldsymbol{\mu}) \otimes (\boldsymbol{x}_i - \boldsymbol{\mu}) \right] .$$

Taking the derivative with respect to $\text{vec}\boldsymbol{\Sigma}$, we get the Jacobian,

$$\boldsymbol{J} = \frac{1}{n} \sum_{i=1}^{n} \kappa'_\beta \left[(\boldsymbol{x}_i - \boldsymbol{\mu})^\top \boldsymbol{\Sigma}^{-1} (\boldsymbol{x}_i - \boldsymbol{\mu}) \right] \text{vec} \left[(\boldsymbol{x}_i - \boldsymbol{\mu}) \otimes (\boldsymbol{x}_i - \boldsymbol{\mu}) \right] \text{vec} \left[\boldsymbol{\Sigma}^{-1} (\boldsymbol{x}_i - \boldsymbol{\mu}) (\boldsymbol{x}_i - \boldsymbol{\mu})^\top \boldsymbol{\Sigma}^{-1} \right]$$

Then taking the

$$\text{tr} (\boldsymbol{J}) = \frac{1}{n} \sum_{i=1}^{n} \kappa'_\beta \left[(\boldsymbol{x}_i - \boldsymbol{\mu})^\top \boldsymbol{\Sigma}^{-1} (\boldsymbol{x}_i - \boldsymbol{\mu}) \right] \left[(\boldsymbol{x}_i - \boldsymbol{\mu})^\top \boldsymbol{\Sigma}^{-1} (\boldsymbol{x}_i - \boldsymbol{\mu}) \right]^2 = \frac{1}{n} \sum_{i=1}^{n} \kappa'_\beta (r_i) \, r_i^2$$

where $r_i = (\boldsymbol{x}_i - \boldsymbol{\mu})^\top \boldsymbol{\Sigma}^{-1} (\boldsymbol{x}_i - \boldsymbol{\mu})$. The function, $\kappa'_\beta(r)\, r^2$, has stationary points equal to the three roots from the following polynomial

$$(\beta^2 - 64)d^3 - 3[16/3 + (r-2)\beta](\beta+8)d^2 + 3(r^2 - 2r + 8/3)(\beta+8)\beta d - \beta^2 r^3, \qquad \text{(C.13)}$$

and if r_1, r_2 & r_3 are the roots then we have

$$K = \max\left\{\left|\kappa'_\beta(r_1)\, r_1^2\right|, \left|\kappa'_\beta(r_2)\, r_2^2\right|, \left|\kappa'_\beta(r_3)\, r_3^2\right|\right\}. \qquad \text{(C.14)}$$

Figure C.1 illustrates the function, stationary points and bounds when $d = 2$ & 8 and $\beta = 4d(d+2)/(d+4)$.

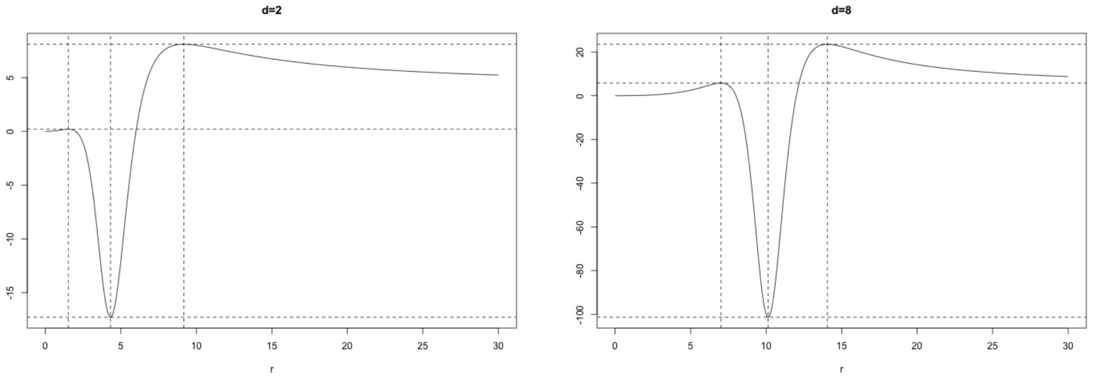

Figure C.1: The function, $\kappa'_\beta(r)\, r^2$, when $d = 2$ (left) & $d = 8$ (right). Both function have $\beta = 4d(d+2)/(d+4)$. Highlighted are the stationary points and maximum and minimum.

To illustrate the log-likelihoods fluctuations, we generated two samples of size 100 from (C.16) with $d = 10$ and $d = 16$. Then we use either (C.12) or (C.15) with $a = 1$ to estimate $\boldsymbol{\Sigma}$. Figure C.2 shows the fluctuations in the log-likelihood sequences. This motivates the use of the MM updates or create an alternative to the fixed point update given in (C.12).

The function are because the function $\kappa_\beta(r)$ is Lipschitz continuous with constant $K > 1$ so a

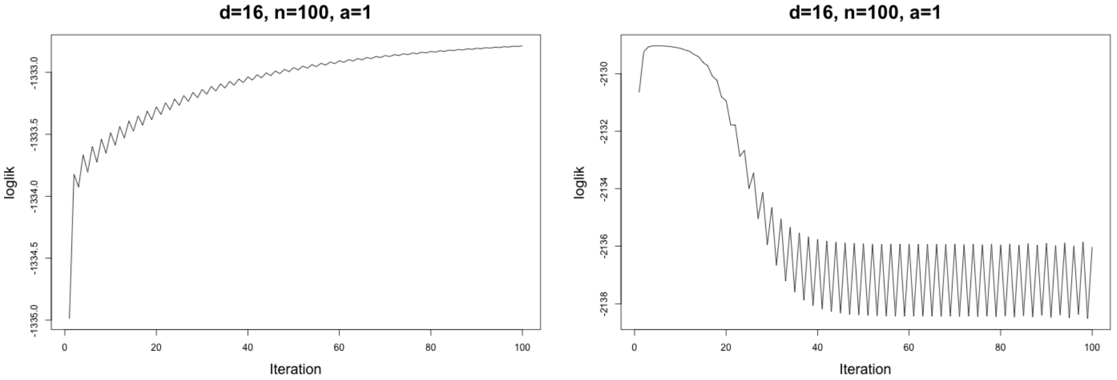

Figure C.2: Two illustrations with log-likelihoods fluctuations using (C.12) or (C.15) with $a = 1$. The left panel has $d = 10$ and the right panel has $d = 16$.

natural fix is a relaxed Picard fixed point iteration

$$\boldsymbol{\Sigma}^{(q+1)} = (1-a)\,\boldsymbol{\Sigma}^{(q)} + a\boldsymbol{M}\left(\boldsymbol{\Sigma}^{(q)}\right). \tag{C.15}$$

If $a < 1/K$ the fixed point iteration will converge. However, it seems the $a = 1/K$ is too strict of a bound and simulations show it can be can be higher. Figure C.3 show $a = 1/K$ as function of d.

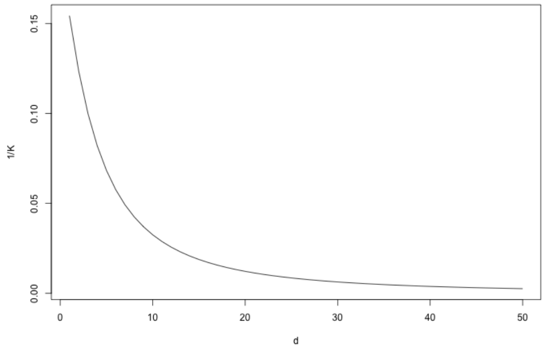

Figure C.3: A plot of $1/K$ as function of d when $\beta = 4d(d+2)/(d+4)$.

To provide evidence that the weight can be larger we use the fixed point update (C.15) for a single component while varying the dimension and the weight. We generate $n = 100$ observations

from a single component of MLN using

$$\boldsymbol{\mu} = \mathbf{0}_d,\ \boldsymbol{\Sigma} = \boldsymbol{I}_d,\ \beta = \frac{4d(d+2)}{(d+4)} \tag{C.16}$$

and only update $\boldsymbol{\Sigma}$. For higher dimensions we also examine update where we constraint $\boldsymbol{\Sigma}$ to be diagonal. For each sample generated we initialize at the true value given in (C.16) then for each weight $a = 0.5, \ldots, 1$, we perform 100 EM iterations. Then we check the log-likelihood sequence for fluctuations by examining the minimum difference between the log-likelihood values. If this difference is negative then we consider this an unstable or fluctuated run.

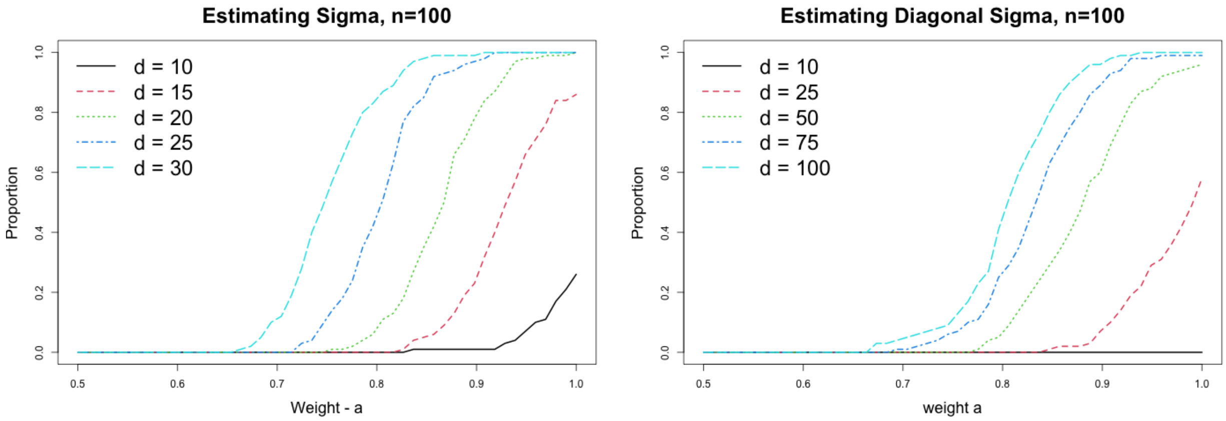

Figure C.4: Plots the proportion of runs with fluctuations while varying the weight, $a = 1$ in (C.15) while varying the number of variables d and having the sample size equal to $n = 100$. The left panel involves estimating a general $\boldsymbol{\Sigma}$ and the middle panel involves estimating a diagonal $\boldsymbol{\Sigma}$.

Figures C.4 and C.5 indicate that the weight does not need to as strict or equal to $1/K$ (the value of $1/K$ is show in Figure C.3). Browne (2022) suggests using the weight by given by

$$a = \frac{1}{1 + \beta\frac{d+4}{4d(d+2)}}, \tag{C.17}$$

so that the weight is 1 when $\beta = 0$ and the weight is $1/2$ when β is equal to the largest possible value of $4d(d+2)/(d+4)$.

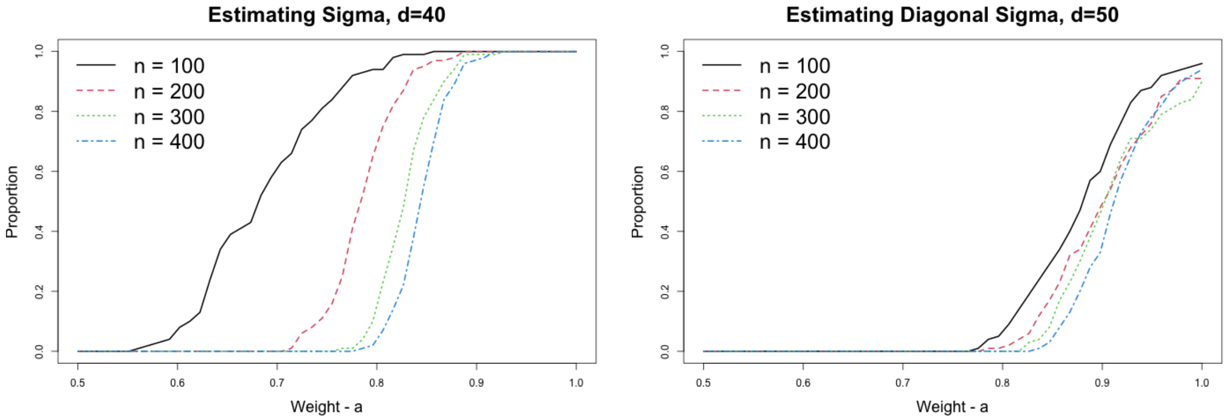

Figure C.5: Plots the proportion of runs with fluctuations while varying the weight, $a = 1$ in (C.15) while varying the number of variables d and having the sample size equal to $n = 100$. The left panel involves estimating a general $\mathbf{\Sigma}$ and the middle panel involves estimating a diagonal $\mathbf{\Sigma}$.

We illustrate using these two different weights in Figure C.6. This Figure uses the same data as Figure C.2 but now we have added the log-likelihood sequences produced when using the weights from (C.14) and (C.17). The update using (C.17) convergences faster and preserves monotonicity.

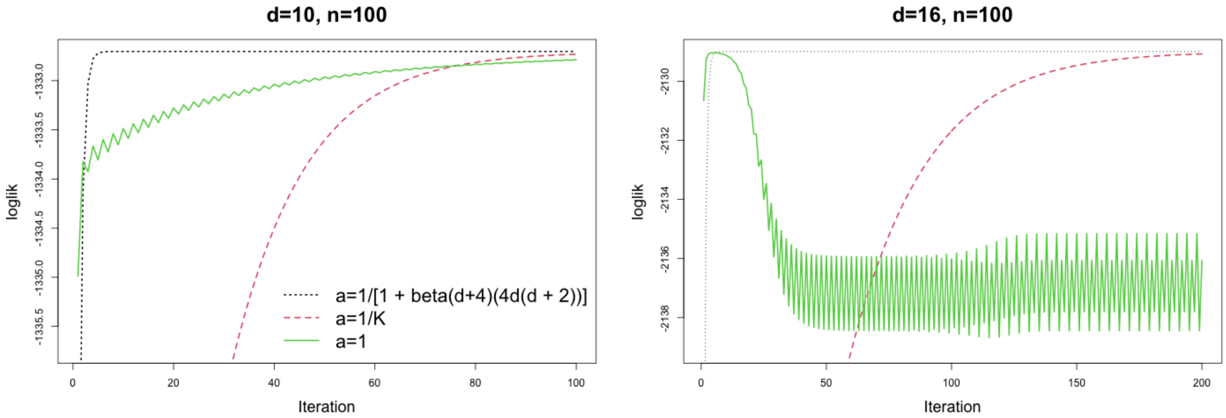

Figure C.6: Two illustrations with and without log-likelihoods fluctuations using different weights. The left panel has $d = 10$ and the right panel has $d = 16$.

We further compare the weights by examining the log-likelihood sequences produced when using a weight equal to $1/K$ or given (C.17) while checking each sequence for log-likelihood fluctuations.

We generate 100 observations while varying d to be $10, 15, 20$ and 25 from $(C.16)$ or instead using $\mathbf{\Sigma} = \boldsymbol{I}_d(1-0.95)+0.95\boldsymbol{J}_d$ where $\boldsymbol{J}_d$ is a $d\times d$ matrix of ones. Table C shows the average number of iterations until convergence while estimating either $\mathbf{\Sigma}$ or a diagonal $\mathbf{\Sigma}$ based on 100 replications for each d. The convergence tolerance was set to 10^{-4}. Both weights yielded monotonic log-likelihood sequences and using (C.17) requires less iterations.

	$\mathbf{\Sigma} = \boldsymbol{I}_d$ Estimating $\mathbf{\Sigma}$		$\mathbf{\Sigma} = \boldsymbol{I}_d$ Estimating Diagonal $\mathbf{\Sigma}$		$\mathbf{\Sigma} = \boldsymbol{I}_d(1-0.95)+0.95\boldsymbol{J}_d$ Estimating $\mathbf{\Sigma}$	
$a =$	$1/\left[1+\beta\frac{d+4}{4d(d+2)}\right]$	$1/K$	$1/\left[1+\beta\frac{d+4}{4d(d+2)}\right]$	$1/K$	$1/\left[1+\beta\frac{d+4}{4d(d+2)}\right]$	$1/K$
$d = 10$	18.4	1405.8	9.6	788.8	21.7	1640.6
15	13.7	2284.1	10.7	1745.1	14.3	2331.6
20	12.7	3614.8	11.1	2991.2	13.0	3633.8
25	12.1	5490.9	12.1	4681.3	12.2	5487.9

Table C.1: Average number of iterations until convergence with a tolerance equal to 10^{-4} based on 100 replications, sample size equal to 100.

D Simulation study: comparing MM and FP algorithms

Table D.2 completes the simulation study described within Section 7.1 of the manuscript by giving the results in the case $d = 16$.

	Mean from (FP, MM) of					
	Time		Iterations		$\hat{l}_{\max} - \hat{l}$	
EVV	0.062	2.196	48	1000	0.0	107.5
VEV	0.151	2.202	106	1000	0.0	103.9
EEV	0.190	2.137	135	1000	2.1	7.8
EVE	2.395	2.085	966	1000	0.0	94.9
VVE	2.367	2.052	961	1000	0.0	10.7
VVV	0.054	0.056	42	27	20.5	0.0
EEE	0.042	0.055	35	31	0.3	0.0
VVI	0.015	0.035	17	31	0.0	0.0
EEI	0.011	0.026	12	22	0.0	0.0
VEE	0.045	0.013	35	6	0.0	71.6
VII	0.007	0.016	6	14	0.0	0.0
VEI	0.012	0.017	13	14	0.0	7.6
EVI	0.015	0.016	16	12	0.0	15.2
EII	0.006	0.014	6	12	0.0	0.0

Table D.2: Average computational time and number of iterations until convergence (with the maximum number of iterations fixed to 1000), for each estimation algorithm over 100 replications when $d = 16$. The columns with header $\hat{l}_{\max} - \hat{l}$ show the average difference between the log-likelihood at convergence and the maximum log-likelihood for that data set. The output is displayed in pairs (FP, MM), which is the result from the FP and MM algorithms.

E Simulation study: Investigating some aspects of the MLN mixture

An expanded form for the parameter setups given in Equations (16)–(18) of the paper is given by

$$\boldsymbol{\theta}_2(\delta,\lambda,\rho,d)=\left\{\pi_j=\frac{1}{2},\begin{bmatrix}\boldsymbol{\mu}_1\\ \boldsymbol{\mu}_2\end{bmatrix}=\delta\begin{bmatrix}-\mathbf{1}_d\\ \mathbf{1}_d\end{bmatrix},\begin{bmatrix}\boldsymbol{\Sigma}_1\\ \boldsymbol{\Sigma}_2\end{bmatrix}=\begin{bmatrix}\lambda\boldsymbol{\Xi}_d(\rho)\\ \frac{1}{\lambda}\boldsymbol{\Xi}_d(\rho)\end{bmatrix},\beta_j=\frac{4d(d+2)}{(d+4)}\right\},$$

$$\boldsymbol{\theta}_3(\delta,\lambda,\rho,d)=\left\{\pi_j=\frac{1}{3},\begin{bmatrix}\boldsymbol{\mu}_1\\ \boldsymbol{\mu}_2\\ \boldsymbol{\mu}_3\end{bmatrix}=\delta\begin{bmatrix}-\mathbf{1}_{d/2}\\ \mathbf{0}_{d/2}\\ +\mathbf{1}_{d/2}\end{bmatrix},\begin{bmatrix}\boldsymbol{\Sigma}_1\\ \boldsymbol{\Sigma}_2\\ \boldsymbol{\Sigma}_3\end{bmatrix}=\begin{bmatrix}\lambda\boldsymbol{\Xi}(\rho)\\ \boldsymbol{\Xi}(\rho)\\ \frac{1}{\lambda}\boldsymbol{\Xi}(\rho)\end{bmatrix},\ \beta_j=\frac{4d(d+2)}{(d+4)}\right\},$$

and

$$\boldsymbol{\theta}_4(\delta,\lambda,\rho,d)=\left\{\pi_j=\frac{1}{4},\begin{bmatrix}\boldsymbol{\mu}_1^\top\\ \boldsymbol{\mu}_2^\top\\ \boldsymbol{\mu}_3^\top\\ \boldsymbol{\mu}_4^\top\end{bmatrix}=\delta\begin{bmatrix}-\mathbf{1}_{d/2}^\top & -\mathbf{1}_{d/2}^\top\\ \mathbf{1}_{d/2}^\top & -\mathbf{1}_{d/2}^\top\\ -\mathbf{1}_{d/2}^\top & \mathbf{1}_{d/2}^\top\\ \mathbf{1}_{d/2}^\top & \mathbf{1}_{d/2}^\top\end{bmatrix},\begin{bmatrix}\boldsymbol{\Sigma}_1\\ \boldsymbol{\Sigma}_2\\ \boldsymbol{\Sigma}_3\\ \boldsymbol{\Sigma}_4\end{bmatrix}=\begin{bmatrix}\frac{1}{\lambda}\boldsymbol{\Xi}(\rho)\\ \boldsymbol{\Xi}(\rho)\\ \boldsymbol{\Xi}(\rho)\\ \lambda\boldsymbol{\Xi}(\rho)\end{bmatrix},\ \beta_j=\frac{4d(d+2)}{(d+4)}\right\}.$$

E.1 Asymptotic properties of the ML estimators

Here, we present the additional figures from the simulation study described within Section 8.1 of the manuscript.

Figures E.7, E.8, and E.9 show the $\log(\text{Avarage MSE})$ when varying the data generating and fitted models for $k = 2$, 3, and 4, respectively. Each column is related to the data generating model and each row to the fitted model. Each cell contains the $\log(\text{Avarage MSE})$ based on 100 replications when $d \in \{2, 8, 16\}$ and the sample size varies by color within the set $\{100, 500, 1000\}$.

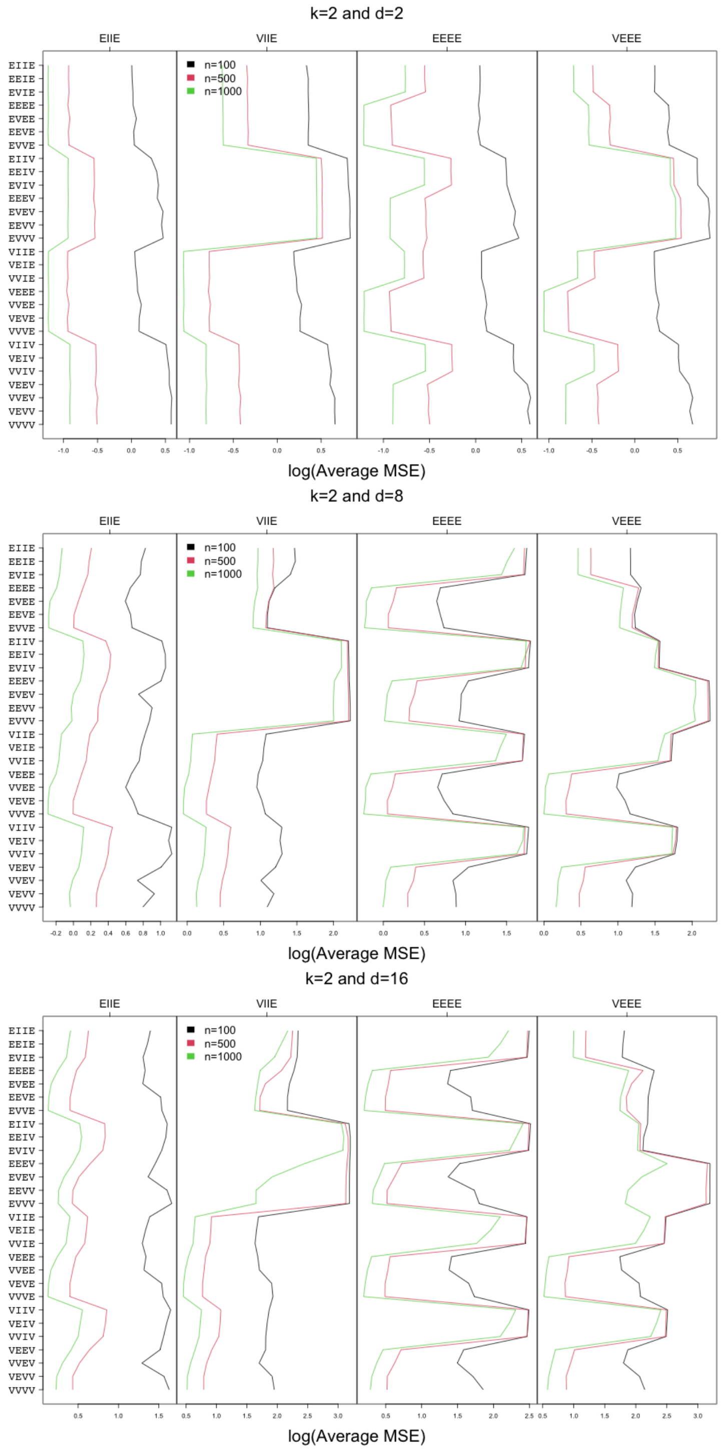

Figure E.7: The log (Avarage MSE) when varying the data generating and fitted models. In each subplot, columns refer to the data generating model and rows to the fitted model. Each point on the line represents the log (Avarage MSE), based on 100 replications, when $k = 2$, $d \in \{2, 8, 16\}$, and $n \in \{100, 500, 1000\}$.

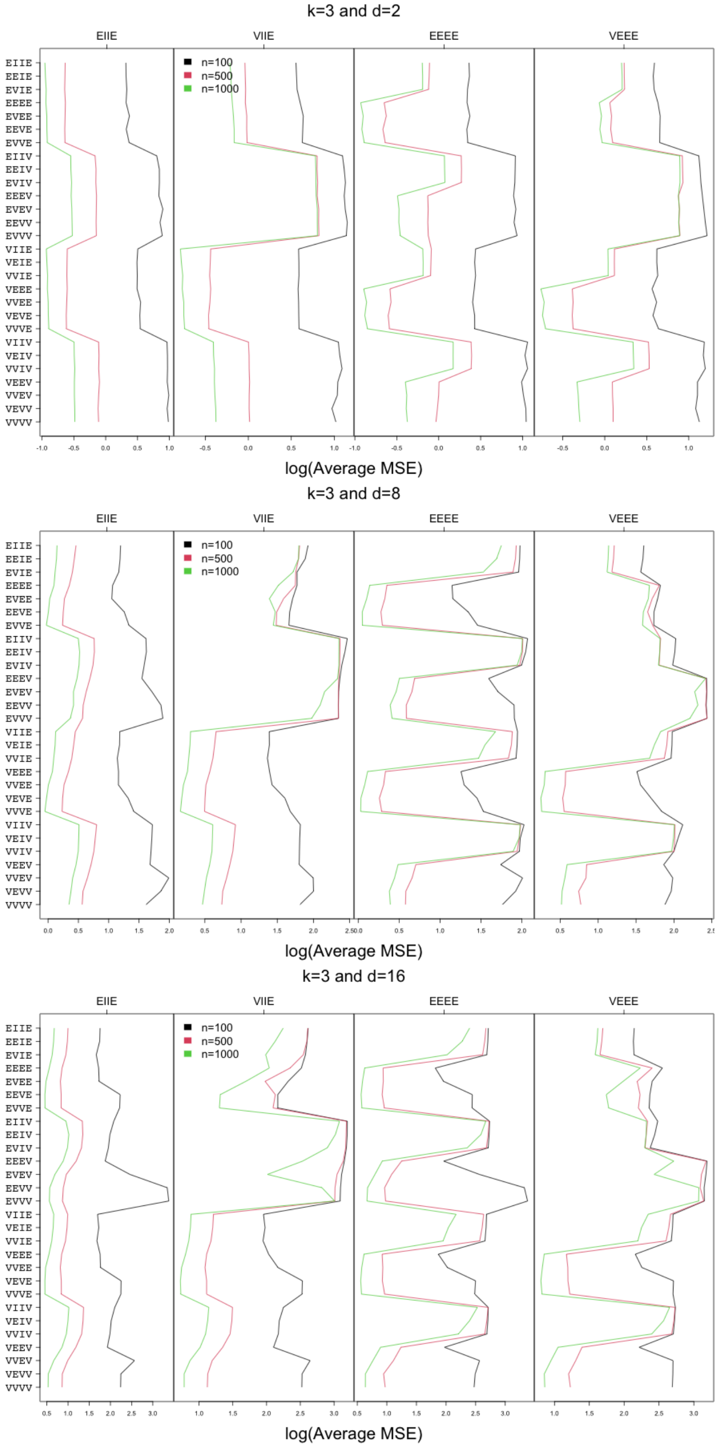

Figure E.8: The log (Avarage MSE) when varying the data generating and fitted models. In each subplot, columns refer to the data generating model and rows to the fitted model. Each point on the line represents the log (Avarage MSE), based on 100 replications, when $k = 3$, $d \in \{2, 8, 16\}$, and $n \in \{100, 500, 1000\}$.

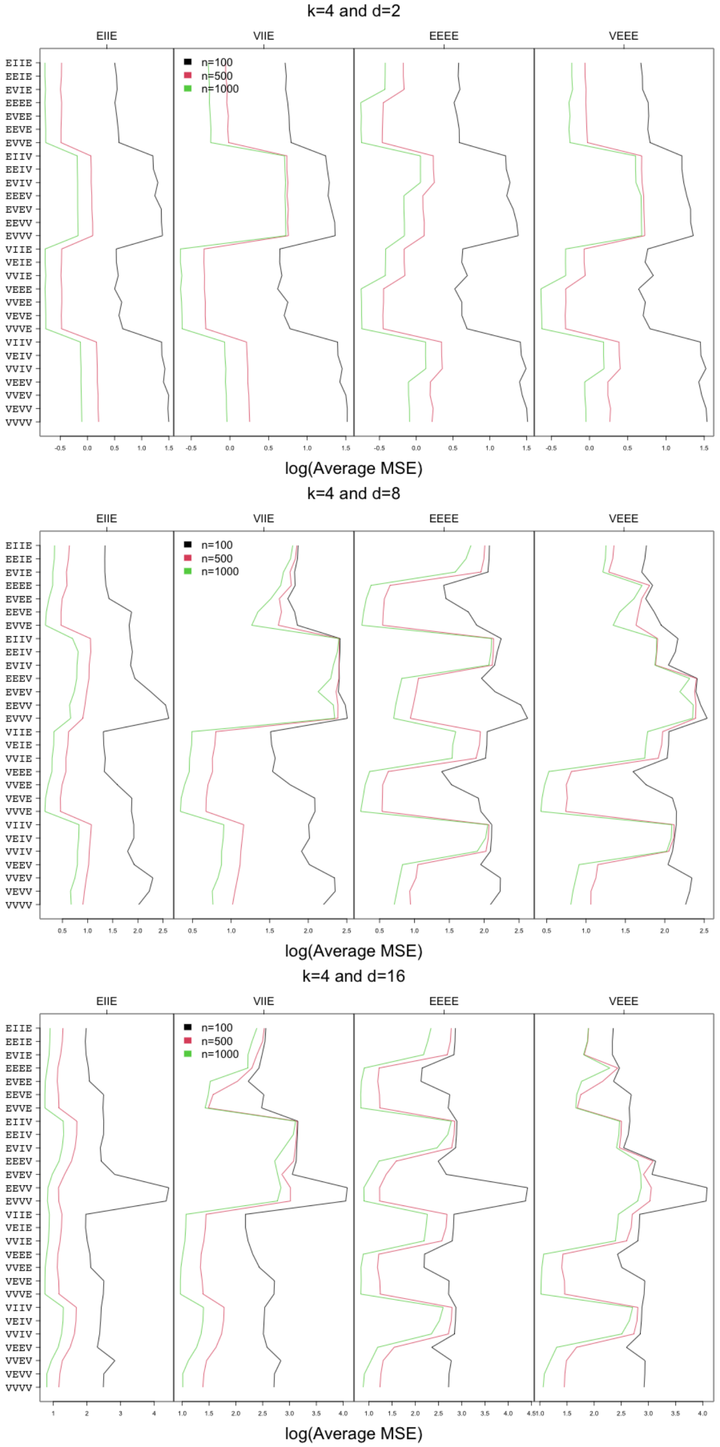

Figure E.9: The log (Avarage MSE) when varying the data generating and fitted models. In each subplot, columns refer to the data generating model and rows to the fitted model. Each point on the line represents the log (Avarage MSE), based on 100 replications, when $k = 4$, $d \in \{2, 8, 16\}$, and $n \in \{100, 500, 1000\}$.

E.2 Choosing the number of components

Here, we present the additional tables from the simulation study described within Section 8.2 of the manuscript.

Tables E.3, E.5, and **??** give the frequency table of the number of times a covariance model was selected using the BIC when $k = 2, 3, 4$, respectively. Each column represents a summary of 100 data sets generated from $\theta_k(\delta, \lambda, \rho, d)$ using $k = 4$ & $\delta = 3$ and varying $d \in \{2, 8, 16\}$, $\rho \in \{0, 0.5\}$, $\lambda \in \{1, 2\}$, and $n \in \{50, 100\}$.

	Data generated from model with (λ,ρ) & $d=2$								Data generated from model with (λ,ρ) & $d=8$								Data generated from model with (λ,ρ) & $d=16$							
	$(1,0)$		$(2,0)$		$(1,0.5)$		$(2,0.5)$		$(1,0)$		$(2,0)$		$(1,0.5)$		$(2,0.5)$		$(1,0)$		$(2,0)$		$(1,0.5)$		$(2,0.5)$	
n	50	100	50	100	50	100	50	100	50	100	50	100	50	100	50	100	50	100	50	100	50	100	50	100
VIIE	10	2	2		84	93	16		5	6	2		100	99	6		6	4	11		96	100	16	
EIIE	79	90	14		2		2		95	94	3						94	96	8					
VEEE			2	2	1		56	95			7	4			48	87							1	4
EEEE			63	86			5				49	90							2	6				
VVEE			2	1	1		7	3			8	1			33	11			9	9			50	78
EVEE			6	3							28	5							30	76				
VVEV															1				7	1			32	17
EVEV											2								33	8				
VEEV			1				1								11	2								1
EEVE			7	7			1																	
EEIE	5	7			2																			
VEVE			1		1		10	2																
VIIV					2	3	1							1	1						4		1	
VEIE					3	2	1																	
EIIV	2	1	1		2																			
EVIE	4																							
EEEV			1	1							1													
VVIE					2																			
VEIV						1																		
VVIV						1																		
VVVE																								
EVVE																								
EEIV																								
EVIV																								
EEVV																								
VEVV																								
VVVV																								
EVVV																								

Table E.3: Number of times each parsimonious model was selected by the BIC. Each column represents a summary of 100 data sets generated from a MLN mixture model with $\boldsymbol{\theta}_k\left(\delta,\lambda,\rho,d\right)$ using $k=2$ & $\delta=3$ and varying $d\in\{2,8,16\}$, $\rho\in\{0,0.5\}$, $\lambda\in\{1,2\}$ and $n\in\{50,100\}$.

	Data generated from model with (λ,ρ) & $d=2$								Data generated from model with (λ,ρ) & $d=8$								Data generated from model with (λ,ρ) & $d=16$							
	$(1,0)$		$(2,0)$		$(1,0.5)$		$(2,0.5)$		$(1,0)$		$(2,0)$		$(1,0.5)$		$(2,0.5)$		$(1,0)$		$(2,0)$		$(1,0.5)$		$(2,0.5)$	
n	50	100	50	100	50	100	50	100	50	100	50	100	50	100	50	100	50	100	50	100	50	100	50	100
EIIE	77	88	30	7	30	6	9	4	96	99	7				1		92	100	31					
VIIE	4	6	2		42	81	14	3	3	1	3		96	100	24		6		26	1	92	100	52	
EEEE	1	2	55	85	3	1	20	10			40	93			3				13	7			1	
VEEE	1		2		2	1	28	67			9	3			29	93			2				9	4
VVEE	1			1			3	2			9				27	4			1	22			3	70
EVEE			3	1	3		7	4			27	4			4				6	54			6	
VIIV													4						4		8		18	
VVEV															5				1	2			2	23
EEVE	3		7	6	1	2	6																	
EVEV	1							1			3				1				4	13			2	
VEVE					2	2	7	7																
VEEV															6	3							5	3
EEEV								1			2								10	1			2	
EEIE	8	3			1		2																	
VEIE	1				5	3	1																	
EVIE	2				5	2	1																	
EIIV	1				2				1								2		2					
VVVE					1		1	1																
EVVE			1		1		1																	
VVIE					1	1																		
EVVV		1			1																			
VVVV						1																		
EEIV																								
VEIV																								
EVIV																								
VVIV																								
EEVV																								
VEVV																								

Table E.4: Number of times each parsimonious model was selected by the BIC. Each column represents a summary of 100 data sets generated from a MLN mixture model with $\boldsymbol{\theta}_k\,(\delta,\lambda,\rho,d)$ using $k=3$ & $\delta=3$ and varying $d\in\{2,8,16\}$, $\rho\in\{0,0.5\}$, $\lambda\in\{1,2\}$ and $n\in\{50,100\}$.

	Data generated from model with (λ, ρ) & $d = 2$							
	$(1, 0)$		$(2, 0)$		$(1, 0.5)$		$(2, 0.5)$	
n	50	100	50	100	50	100	50	100
EIIE	89	93	22	3	52	31	12	1
VIIE	3				31	63	14	3
EEEE	2		68	95	3	1	45	37
VEEE			1		1		16	57
VVEE				1	1		2	
EVEE	1		6				5	1
VIIV								
EEEV								1
VVEV								
VEEV								
EEIE	5	6			5	3		
EEVE			2	1	1		4	
VEVE					1		1	
EVEV								
VEIE			1		3	1		
EVIE		1			1			
VVIE					1	1		
EIIV								
EVVE							1	
EEVV								
VVVE								
EEIV								
VEIV								
EVIV								
VVIV								
VEVV								
VVVV								
EVVV								

	Data generated from model with (λ, ρ) & $d = 8$							
	$(1, 0)$		$(2, 0)$		$(1, 0.5)$		$(2, 0.5)$	
n	50	100	50	100	50	100	50	100
EIIE	96	99	6		2		2	
VIIE	2	1	1		93	98	9	
EEEE			59	98			13	
VEEE			3	2			50	96
VVEE	1		7				15	1
EVEE	1		19				4	
VIIV					5	2		
EEEV			3					
VVEV							2	
VEEV							4	3
EEIE								
EEVE								
VEVE								
EVEV			2				1	
VEIE								
EVIE								
VVIE								
EIIV								
EVVE								
EEVV								
VVVE								
EEIV								
VEIV								
EVIV								
VVIV								
VEVV								
VVVV								
EVVV								

	Data generated from model with (λ, ρ) & $d = 16$							
	$(1, 0)$		$(2, 0)$		$(1, 0.5)$		$(2, 0.5)$	
n	50	100	50	100	50	100	50	100
EIIE	98	100	16				2	
VIIE	2		25		86	96	41	
EEEE			22	9			6	
VEEE			1	1			17	14
VVEE				26				62
EVEE				57				1
VIIV			2		14	4	13	
EEEV			27	1			6	
VVEV				3				22
VEEV			1				13	1
EEIE								
EEVE								
VEVE			4				1	
EVEV				3				
VEIE								
EVIE								
VVIE								
EIIV			2					
EVVE								
EEVV							1	
VVVE								
EEIV								
VEIV								
EVIV								
VVIV								
VEVV								
VVVV								
EVVV								

Table E.5: Number of times each parsimonious model was selected by the BIC. Each column represents a summary of 100 data sets generated from a MLN mixture model with $\boldsymbol{\theta}_k(\delta, \lambda, \rho, d)$ using $k = 4$ & $\delta = 3$ and varying $d \in \{2, 8, 16\}$, $\rho \in \{0, 0.5\}$, $\lambda \in \{1, 2\}$ and $n \in \{50, 100\}$.

Table E.6 is the analogous, when $d = 16$, of Table 8 in the paper, which we recall to refer to the case $d = 2$. Table E.6 gives the number of components selected by the BIC when fixing the model structure and varying the number of components.

Table E.7 is the analogous, when $d = 16$, of Table 9 in the paper, which we recall to refer to the case $d = 2$. Table E.7 gives the number of times each pair (model, k) was selected by the BIC for each scenario. The search space was $k \in \{1, \ldots, 8\}$ and all the possible model structures.

Data generated from $\boldsymbol{\theta}_k\,(\delta, \lambda, \rho, d = 2)$						Selected k $(\tilde{k})$							
k	Model	ρ	λ	δ	n	1	2	3	4	5	6	7	8
2	EIIE	0.0	1	0.25	100	86	14						
2	EIIE	0.0	1	0.25	200	35	65						
2	EIIE	0.0	1	0.25	300	3	97						
2	EEEE	0.5	1	1.25	100	98	2						
2	EEEE	0.5	1	1.25	200	57	43						
2	EEEE	0.5	1	1.25	300	6	94						
2	VIIE	0.0	2	0.25	100		97	3					
2	VIIE	0.0	2	0.25	200		100						
2	VIIE	0.0	2	0.25	300		100						
2	VEEE	0.5	2	1.25	100		98	1	1				
2	VEEE	0.5	2	1.25	200		100						
2	VEEE	0.5	2	1.25	300		100						
3	EIIE	0.0	1	0.75	100		53	47					
3	EIIE	0.0	1	0.75	200			100					
3	EIIE	0.0	1	0.75	300			100					
3	EEEE	0.5	1	3.00	100	98	1	1					
3	EEEE	0.5	1	3.00	200	63	3	34					
3	EEEE	0.5	1	3.00	300	4		96					
3	VIIE	0.0	2	0.75	100		33	66	1				
3	VIIE	0.0	2	0.75	200		1	99					
3	VIIE	0.0	2	0.75	300			100					
3	VEEE	0.5	2	3.00	100	1	12	86	1				
3	VEEE	0.5	2	3.00	200			100					
3	VEEE	0.5	2	3.00	300			100					
4	EIIE	0.0	1	0.50	100	1	28	69	2				
4	EIIE	0.0	1	0.50	200			25	75				
4	EIIE	0.0	1	0.50	300			2	98				
4	EEEE	0.5	1	1.00	100	99	1						
4	EEEE	0.5	1	1.00	200	14			86				
4	EEEE	0.5	1	1.00	300			1	99				
4	VIIE	0.0	2	0.50	100		13	80	7				
4	VIIE	0.0	2	0.50	200			13	87				
4	VIIE	0.0	2	0.50	300				100				
4	VEEE	0.5	2	1.00	100	43	22	2	31	2			
4	VEEE	0.5	2	1.00	200				99	1			
4	VEEE	0.5	2	1.00	300				100				

Table E.6: Number of times each value of k is selected by the BIC when fixing the model structure and varying the number of components. Each row refers to 100 data sets generated from a MLN mixture with $\boldsymbol{\theta}_k\,(\delta, \lambda, \rho, d = 16)$.

Data generated from $\boldsymbol{\theta}_k$ $(\delta, \lambda, \rho, d = 2)$						Selected k					Selected fitted model					When $k = \tilde{k}$ and	
k	Model	λ	ρ	δ	n	1	2	3	4	≥ 5	EIIE	VIIE	VEEE	EEEE	VVEE	Model	Subset
2	EIIE	1	0.0	0.2	100	83	17				100					17	17
2	EIIE	1	0.0	0.2	200	28	72				98	2				70	72
2	EIIE	1	0.0	0.2	300	1	99				98	2				97	99
2	EEEE	1	0.5	1.2	100			2	14	84	86	9		1			
2	EEEE	1	0.5	1.2	200	3		3		94	84	7		3			
2	EEEE	1	0.5	1.2	300	4	16	1		79	73	4	9	5		8	11
2	VIIE	2	0.0	0.2	100	18	77	5			19	74				72	73
2	VIIE	2	0.0	0.2	200		99	1				99				98	98
2	VIIE	2	0.0	0.2	300		100					100				100	100
2	VEEE	2	0.5	1.2	100		10	13	19	58	1	62		14	1	1	9
2	VEEE	2	0.5	1.2	200		38	7	3	52		46		33	10	10	38
2	VEEE	2	0.5	1.2	300		84			16		15		33	50	50	83
3	EIIE	1	0.0	0.8	100		51	49			93	3				49	49
3	EIIE	1	0.0	0.8	200		3	97			100					97	97
3	EIIE	1	0.0	0.8	300			100			100					100	100
3	EEEE	1	0.5	3.0	100	18	41	4	2	35	32	2	1	31			2
3	EEEE	1	0.5	3.0	200	23	56	11	3	7	7		7	44	5	4	6
3	EEEE	1	0.5	3.0	300	3	18	77	2				70	18	1	70	71
3	VIIE	2	0.0	0.8	100		23	77				86				72	72
3	VIIE	2	0.0	0.8	200			100				100				100	100
3	VIIE	2	0.0	0.8	300			100				99				99	99
3	VEEE	2	0.5	3.0	100		39	41	9	11		9		57			27
3	VEEE	2	0.5	3.0	200		1	89	10					31	61	61	86
3	VEEE	2	0.5	3.0	300			95	4	1				15	82	82	95
4	EIIE	1	0.0	0.5	100		27	69	4		92	7				4	4
4	EIIE	1	0.0	0.5	200			31	69		97	3				69	69
4	EIIE	1	0.0	0.5	300			1	99		99	1				98	99
4	EEEE	1	0.5	1.0	100	26	21	12	7	34	29	3	2	24	2		3
4	EEEE	1	0.5	1.0	200	3	2	4	84	7	5		79	5	1	77	79
4	EEEE	1	0.5	1.0	300			10	89	1			86	3		85	86
4	VIIE	2	0.0	0.5	100		14	70	15	1		94				15	15
4	VIIE	2	0.0	0.5	200			8	92			97				90	90
4	VIIE	2	0.0	0.5	300				100			100				100	100
4	VEEE	2	0.5	1.0	100		32	20	17	31	2	25		31			6
4	VEEE	2	0.5	1.0	200				94	6				7	87	86	90
4	VEEE	2	0.5	1.0	300				99	1				1	99	99	99

Table E.7: Counts of the BIC choices, in the case $d = 16$, when the search is over $k = 1, \ldots, 8$ and all or some of the parsimonious structures. There are four blocks of columns. In the first one, there are the parameters/quantities used to generated the 100 data sets. From the second block onwards, there is the number of times: each value of k is picked (2nd block), each model is selected (3rd block), and each pair (model, k) is selected (4nd block), where "model" belongs to the whole family, in the 2nd last column, and to the subset of models of the 3rd block in the last column.

F Simulation study: Computational time

To quantify the viability of the estimation procedure on large data, we partition the models into two groups, the first obtained by fixing the rotation matrices to be the identity matrix, and the second leaving the rotations matrices to be estimated form the data. The two groups refer to the third letter in the four letter notation; the first group has "I", while the other has either "E" or "V". We will denote these two groups with $\mathcal{I}$ and $\overline{\mathcal{I}}$. In detail,

- the set $\mathcal{I}$ contains the models EIIE, VIIE, EEIE, VEIE, EVIE, VVIE, EIIV, VIIV, EEIV, VEIV, EVIV, and VVIV,
- while the set $\overline{\mathcal{I}}$ contains the models EEEE, EEVE, VEVE, VVVE, EVEE, VVEE, VEEE, EVVE, EEEV, EEVV, VEVV VVVV, EVEV, VVEV, VEEV, and EVVV.

As the members of $\mathcal{I}$ have less parameters than the members of $\overline{\mathcal{I}}$, we consider different number of variables for each set. For $\overline{\mathcal{I}}$ we consider $d = \{16, 24, 32, 40\}$, with the parameters equal to $\boldsymbol{\theta}_3\,(\delta = 5, \lambda = 1, \rho = 0, d)$ and sample size equal to 2000. For $\mathcal{I}$ we consider $d = \{50, 100, 150, 200\}$, with the parameters equal to $\boldsymbol{\theta}_4\,(\delta = 5, \lambda = 1, \rho = 0, d)$ and sample size equal to 500. For both sets we generate 100 data sets and collect the computational required for 100 iterations. The results are shown in Figure F.10 in terms of average computational times. As expected, regardless of the considered parsimonious structure, the computational time roughly increases with d.

References

Absil, P.-A., R. Mahony, and R. Sepulchre (2008). *Optimization Algorithms on Matrix Manifolds.* Princeton, NJ: Princeton University Press.

Anderson, E., Z. Bai, C. Bischof, S. Blackford, J. Demmel, J. Dongarra, J. Du Croz, A. Greenbaum, S. Hammarling, A. McKenney, and D. Sorensen (1999). *LAPACK Users' Guide* (Third ed.). Philadelphia, PA: Society for Industrial and Applied Mathematics.

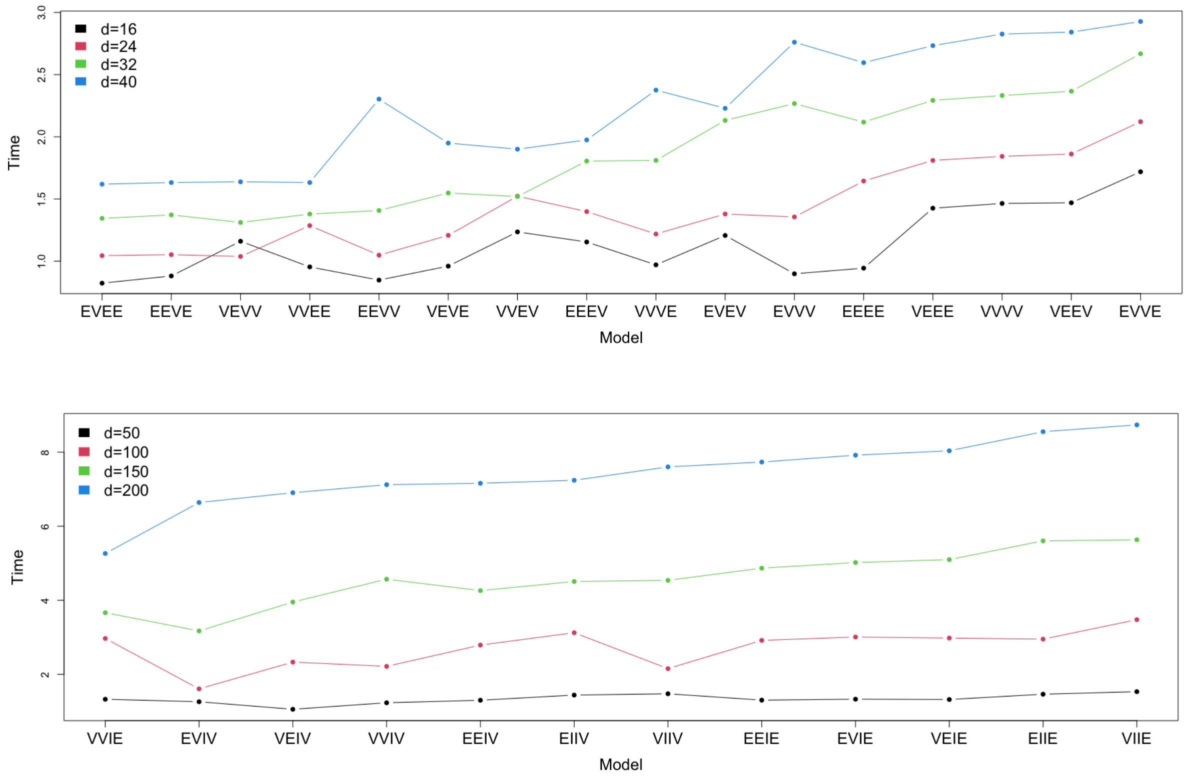

Figure F.10: Average computational time, over 100 replications, to fit the parsimonious MLN mixtures when varying the number of variables (top panel for $\overline{\mathcal{I}}$, bottom panel for $\mathcal{I}$).

Browne, R. and P. McNicholas (2014a). Estimating common principal components in high dimensions. *Advances in Data Analysis and Classification 8*(2), 217–226.

Browne, R. and P. McNicholas (2014b). Orthogonal stiefel manifold optimization for eigen-decomposed covariance parameter estimation in mixture models. *Statistics and Computin 24*(2), 203–210.

Browne, R. P. (2022). Revitalizing the multivariate elliptical leptokurtic-normal distribution and its application in model-based clustering. *Statistics and Probability Letters*.

Cliff, N. (1996). Orthogonal rotation to congruence. *Psychometrika 31*, 33–42.

Kiers, H. (2002). Setting up alternating least squares and iterative majorization algorithms for solving various matrix optimization problems. *Computational Statistics and Data Analysis 41*, 157–170.

Laub, A. (1979, December). A schur method for solving algebraic Riccati equations. *IEEE Transactions on Automatic Control 24*(6), 913–921.

Nocedal, J. and S. J. Wright (2006). *Numerical Optimization* (2e ed.). New York, NY, USA: Springer.

Wonham, W. M. (1968). On a matrix Riccati equation of stochastic control. *SIAM Journal on Control 6*(4), 681–697.
